# Misconceptions in the use of the General Linear Model applied to functional MRI: a tutorial for junior neuro-imagers

**DOI:** 10.3389/fnins.2014.00001

**Published:** 2014-01-21

**Authors:** Cyril R. Pernet

**Affiliations:** Brain Research Imaging Centre, Imaging Sciences, University of EdinburghEdinburgh, UK

**Keywords:** GLM, modeling, baseline, derivatives, percentage signal change, fMRI

## Abstract

This tutorial presents several misconceptions related to the use the General Linear Model (GLM) in functional Magnetic Resonance Imaging (fMRI). The goal is not to present mathematical proofs but to educate using examples and computer code (in Matlab). In particular, I address issues related to (1) model parameterization (modeling baseline or null events) and scaling of the design matrix; (2) hemodynamic modeling using basis functions, and (3) computing percentage signal change. Using a simple controlled block design and an alternating block design, I first show why “baseline” should not be modeled (model over-parameterization), and how this affects effect sizes. I also show that, depending on what is tested; over-parameterization does not necessarily impact upon statistical results. Next, using a simple periodic vs. random event related design, I show how the hemodynamic model (hemodynamic function only or using derivatives) can affects parameter estimates, as well as detail the role of orthogonalization. I then relate the above results to the computation of percentage signal change. Finally, I discuss how these issues affect group analyses and give some recommendations.

## Introduction

A common way the analyze functional Magnetic Resonance Imaging (fMRI) time series is to use the General Linear Model (GLM—Friston et al., 1994, 1995; Worsley and Friston, 1995). In short, time series from each voxel (*y*) are analyzed by fitting an experimental design matrix (*X*) in which the different conditions are explicitly described and most often modeled via a convolution by a hemodynamic response function (hrf—Equation 1). Fitting involves finding the parameters (β) that allow scaling each regressor of the experimental design such as to minimize the distance (in the least squares sense) between the data and the model (Equation 2). Having this in mind, it therefore appears essential to have a design matrix (and sampling scheme) that reflects as much as possible of the data.

(1) y=Xβ+ε

*with y the time series from one voxel*, *X the design matrix*, β *the model parameters*, ε *the error (or residuals)*

(2) $$\overset{⌢}{\beta }={\left(X^{T}X\right)}^{-1}X^{T}y$$

(3) $${\hat{\sigma }}^{2}=({\hat{e}}^{T}\hat{e})/(n-rank(X))$$

*with* $\overset{⌢}{\beta }$ *the parameter estimates*, ${\hat{\sigma }}^{2}$ *the variance estimate, and* $\hat{e}$ *the estimated residuals (y–X*$\overset{⌢}{\beta }$)*—note that Equation 2 only applies when *X^T^X* is invertible. When *X^T^X* is rank deficient, a pseudo-inverse is used instead.*

**Figure 1:**
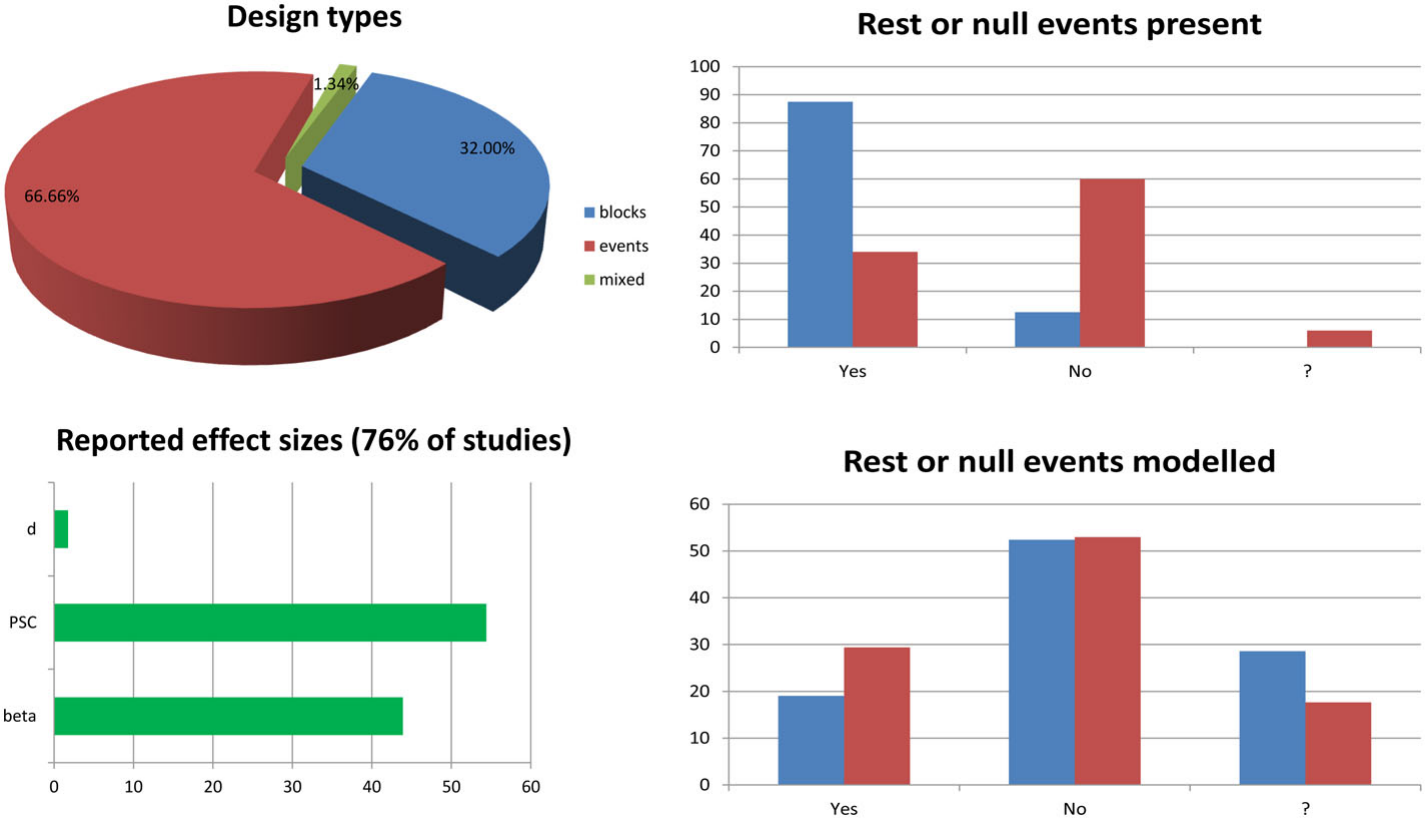
**Survey of 6 months of fMRI literature obtained from the journal Neuroimage between January and June, 2013**. The list of article reviewed and the information sheet used for the survey are presented in annex 1. The pie chart splits studies into block design (*n* = 24.75—blue), event related design (*n* = 50.75—red), and mixed design (*n* = 1.75—green) ones. Bar graphs on the top right show the percentages of studies having rest periods or null events among block design vs. event related design studies. The question mark in the x-axis indicates cases for which it was not clear from the method section if such periods/events were included. Similarly, bar graphs at the bottom right show the percentages of studies modeling these periods or events among studies for which there are present. The bottom left bar graph show the percentages of studies reporting effect sizes in terms of parameter estimates (beta or con), percentage signal change (PSC) or Cohen's d.

While the mathematical machinery behind mass univariate GLM analyses is described in many papers (see e.g., Monti, 2011; Poline and Brett, 2012), and many articles or book chapters present the different type of designs and issues related to sampling and efficiency (Dale, 1999; Friston et al., 1999; Miezin et al., 2000; Birn et al., 2002; Mechelli et al., 2003; Amaro and Barker, 2006; Henson, 2007; Smith et al., 2007), few address in details the issue of modeling the experimental data, i.e., specifying the design matrix, and how this affects results [at the exception of Poline et al. (2007)]. Although most fMRI articles published do model the data appropriately, there are still mistakes and misconceptions about the results from such analyses. One of the most persistent question one can read on forums and discussion lists relate to the modeling of rest periods and/or null events. Here I show that in theory this is better not to model those events, although it does not necessarily impact the statistical results. A short survey of the specialized literature (Figure 1, annex 1) suggests that at least 50% of studies include such periods or events and 23% of them (12% of the total) do model these events. Most studies seemed to have used the right statistical analysis for full brain analysis, but a minority of reported effect sizes might be wrong (for 3 studies of the 75 reviewed, it was not clear if the right parameters were extracted/plotted relative to the statistical maps). Another common issue relates to the use of basis functions and in particular the use of a hemodynamic model and its derivatives. Although there are several advantages in having a more complex model (Lindquist et al., 2009), only ~8% of event related studies used derivatives, and only 2% (1 out of 50 event related studies) used this information at the 2nd level (group) analysis. Importantly, I show that depending on the software and the design, using derivatives have different impact on parameter estimates and users must be aware of differences. Finally, related to both previous issues, is the common question of how to compute percentage signal change in relation to GLM parameters. Most studies report percentage signal change (~54%) in some regions of interest, but none actually described how it was obtained. At best, it is described which software was used [Marsbar (*n* = 5), REX toolbox (*n* = 1), or AFNI (*n* = 3); 12% of cases only] but without specifying the parameter used in those toolboxes. This is a real concern as the reported estimates might be miss-estimated (up to 29% of all studies reviewed), but there is no way to know from the method sections. Here I show how to obtain the percentage signal change using GLM parameters (with derivatives if any) and what should be reported for this metric to be valid.

## Simulation codes and examples

All simulations were programmed in Matlab and the codes can be seen in annexes as well as available to download. The section on Model parameterization corresponds to the file *Model_parameterization.m* (annex 2), the section on Hemodynamic modeling corresponds to the files *Derivative_effect.m* (annex 3), and *Orthogonalization_effect.m* (annex 4) and the section on percentage signal change corresponds to the file *PSC_simulations.m* (annex 5). To run the codes, SPM needs to be installed as well as the function *spm_orth2.m* that can be downloaded. Figures were generated from these codes and post-edited with Photoshop.

## Model parameterization, parameter estimates, and *T*-values

**Figure 2:**
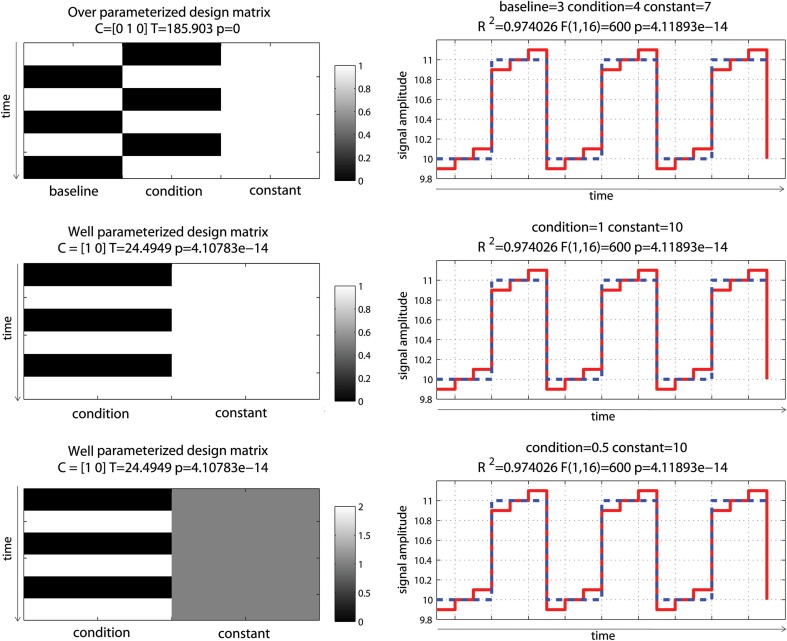
**Controlled block design with a single “active” condition above baseline**. Three different models were used: one over-parameterized one (baseline and condition were modeled—**top**), and two well-parameterized ones (only the condition was modeled) but with the design matrix value either between 0 and 1 **(middle)** or 0 and 2 **(bottom)**. As illustrated on the right hand side, all 3 models (dashed blue lines) fitted the data (red line) the same way giving the same *R*^2^. In addition, since the model degrees of freedom depend on the rank of the design matrix, all models have the same degrees of freedom giving the same *F*- and *p*-values. Differences arise when considering effect sizes (beta values) and *t*-values for the parameter of interest against 0, defined by the contrast C.

Let's consider first a simple controlled block design (one condition of interest—Figure 2). In the simulated data used here, baseline (values 10 ± 0.1) and the condition of interest (values 11 ± 0.1) were alternated such as the “activation blocks” showed 10% signal change on average. Note that the data were created without convolution allowing very simple modeling. The analysis was as follow: (1) model the data with an over-parameterized model (i.e., modeling both baseline and activation); (2) model the data with a well-parameterized model (i.e., modeling activation blocks only); (3) model the data with a well-parameterized model but value range for the regressor of interest in the design matrix equals to 2 rather than 1 (i.e., the regressor of interest in the design matrix was not scaled between 0 and 1—Figure 2). This 3rd model is of particular interest because, depending on the software, the design matrix is not always scaled to 1 and it is essential to understand how this affects parameter estimates. For model 2 and 3, the *t*-values for the condition(s) of interest were computed following Equation 4 and *p*-values obtained from the Student's t distribution.

(4) t=cTβ⌢σ^2cT(XTX)−1c

*c defined the contrast of interest, $\overset{⌢}{\beta }$ are the parameter estimates, ${\hat{\sigma }}^{2}$ is the variance obtained from the residuals, *X* is the design matrix*. For model 1, *X* is rank deficient (because one of the column can be computed with a linear combination of the others), and therefore is *X^T^X* is rank deficient, in which case a pseudo-inverse of *X^T^X* is used instead of the inverse.

Despite different design matrices, all models provided the same fit, i.e., the same fitted data. This is explained by the fact that all design matrices can predict equally well the data. In model 1, the sum of the two first regressors is the constant term; and having this constant term in the model cannot thus change the fit, compared to model 2. The same sums of squares of the effect and the same residuals were therefore obtained, and the amount of variance explained was always the same (same *R*^2^). Also, because degrees of freedom are defined by the rank (i.e., the number of independent regressors) of the design matrix, the over-parameterized model (model 1) had the same degrees of freedom as the other models, and therefore *F*- and *p*-values were also the same.

Differences among models occurred when looking at the parameters of interest: the 1st model returned parameter estimate values different from the simulated data ($\overset{⌢}{\beta }$_1_ = 3 for baseline, $\overset{⌢}{\beta }$_2_ = 4 for activation), whilst model 2 returned parameter estimates that reflected directly the amount of change in the data ($\overset{⌢}{\beta }$_1_ = 1 for activation, $\overset{⌢}{\beta }$_2_ = 10 for baseline/constant). The reason why the estimated parameters in Model 1 do not reflect the simulations is because there is no unique solution, indeed there is an infinite number of possible solutions for the estimated parameter that can lead to the same error (the same sum of square of the error). Following Equations 1 and 2, the data are simply expressed as the sum of weighted regressors plus the error term. Model 2 (i.e., modeling activation only, plus the constant) thus follows Equation 5 and the constant term (the intercept) is given by Equation 6.

(5) $$Y=X_{1}*{\hat{\beta }}_{1}+X_{2}*{\hat{\beta }}_{2}+\hat{e}$$

(6) $$X_{2}*{\hat{\beta }}_{2}=Y-X_{1}*{\hat{\beta }}_{1}-\hat{e}$$

*with Y the data, X the design matrix (*X*_1_ coding for activation and X_2_ coding for the constant term), $\overset{⌢}{\beta }$_1_, $\overset{⌢}{\beta }$_2_, are the parameter estimates and $\hat{e}$ the error*.

It becomes apparent that the constant term (here *X*_2_ * $\overset{⌢}{\beta }$_2_) represents the average across observations of the adjusted data, i.e., the estimated average of the data minus the effect of the activation regressors and the error. In this model, the constant term therefore models baseline, and $\overset{⌢}{\beta }$_1_ reflects the signal change relative to it. In Model 1 (i.e., modeling activation and baseline, plus the constant), individual beta estimate values cannot be interpreted because they are not “estimable”. Since the design matrix is over-parameterized (i.e., *X* is rank deficient), the inverse of *X^T^X* in Equation 2 cannot be obtained, meaning that there is no unique solution. Instead, an infinity of parameter estimate values can be obtained depending on the generalized inverse used (in the code used here, the pinv Matlab function uses the Moore–Penrose pseudo-inverse, giving one, among many, possible solutions). Having different parameter estimate values depending on the method used is, however, not necessarily an issue because the predicted values and the corresponding residuals remain unchanged (as shown above the same model fit is obtained by the different models). This implies that the “right” *T*/*p*-values can be obtained by using a combination of regressors that make their linear dependency irrelevant: in our case, the linear dependency is that “baseline” + “activation” = constant, therefore X*[*k k* –*k*]^*T*^ = 0 (*k* is any constant) and we have *XB* = *X*[*B* + *k*[1 1 –1]^*T*^]. If we are using contrasts orthogonal to [1 1 –1] then our result is independent of the arbitrary constant *k*, and this is an “estimable” contrast. Testing explicitly activation versus baseline (i.e., a contrast [–1 1 0]) in model 1 is one of such contrast, and in this case one obtains the same results as testing activation vs. 0 (i.e., a contrast [1 0]) in model 2 [for a more in depth treatment of this issue see appendix section in Poline and Brett (2012)].

Another important aspect of the GLM is the scale of the design matrix. Since the design matrix is a model of the data, the parameters can be seen as values that simply scale the columns of *X*. A consequence of this is that model 2, for which the activation regressor in the design matrix was scaled between 0 and 1, had a parameter estimate for activation that reflected directly the signal change relative to the constant/baseline ($\overset{⌢}{\beta }$_1_ = 1, $\overset{⌢}{\beta }$_2_ = 10). In contrast, model 3, for which the activation regressor was scaled between 0 and 2, had a parameter estimate for activation of half the value of the signal change ($\overset{⌢}{\beta }$_1_ = 0.5, $\overset{⌢}{\beta }$_2_ = 10). In fMRI, after regressors are convolved by the hemodynamic response model, they are not always rescaled between 0 and 1 and this will matter when looking at the PSC because the parameter estimates do not then reflect directly changes in the signal. However, if we only focus on the statistics, and because *T*-values are defined as the ratio between the parameter estimate and error variance (which is also scaled by the design matrix, see Equation 4), results are identical between different scaled models (here model 2 and 3).

**Figure 3:**
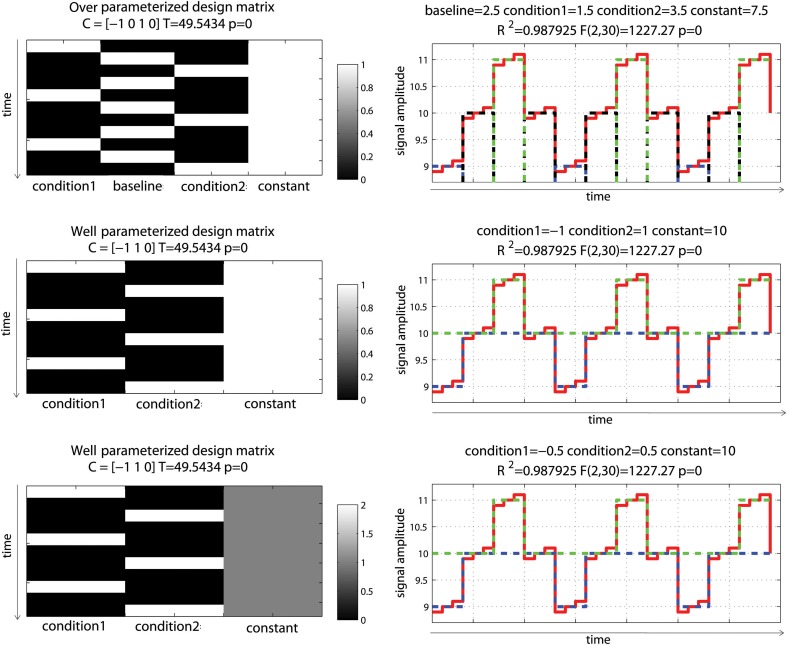
**Alternating block design with two “active” conditions and a baseline**. Three different models were used: one over-parameterized one (condition 1, baseline and condition 2 were modeled—**top**), and two well-parameterized ones (only conditions were modeled) but with the design matrix value either between 0 and 1 **(middle)** or 0 and 2 **(bottom)**. As illustrated on the right hand side, all 3 models fitted the data the same way, giving the same *R*^2^. Parameter estimates however differed. The fitted data for condition 1 are plotted in blue, for condition 2 in red and for the baseline (model 1 only) in black. In model 1, condition 1 and 2 are modeled as positive effects relative to the constant term (7.5 + 1.5 = 9 for condition 1, 7.5 + 3.5 = 11 for condition 2) whereas for model 2 and 3, they are modeled as a negative effect relative to constant for condition 1 (10 – 1 = 9 for model 2 or 10 – 0.5^*^ 2 = 9 for model 3) and a positive effect relative to constant for condition 2 (10 + 1 = 11 for model 2 and 10 + 0.5^*^ 2 = 11 for model 3). Despite those differences, contrasts *C* between the conditions gave the same *T*-values.

Consider now an *alternating block design* (Figure 3). In this simulation, data corresponded to an alternation between two conditions of interest (11 ± 0.1 and 9 ± 0.1) relative to a baseline (10 ± 0.1) having again 10% signal change on average (and thus 20% signal change between the 2 conditions). Doing the same analysis as above, we can observe that all models gave similar fits and that the over-parameterized model (model 1) gave the “wrong” parameter estimates given the data change simulated. However, the contrast between conditions 1 and 2 was always correct. Differences between parameter estimates and standardized variances were identical for model 1 and 2, whilst those values were simply scaled for model 3, such as their ratio (i.e., the *t*-values) gave the same results. This illustrates again that model parameterization does not always impact on statistical results.

These examples illustrate the fundamental point that “contrast specification and the interpretation of results are entirely dependent on the model specification *(and parameterization)* which in turn depends on the design of the experiment” (Poline et al., 2007—italic added). For the reader interested into computational details related to the GLM and application to fMRI, the articles by Monti (2011) and Poline and Brett (2012) are extremely well-documented. For a more comprehensive covering of linear models, a must read is Christensen (2011).

## Hemodynamic modeling and the use of basis functions

**Figure 4:**
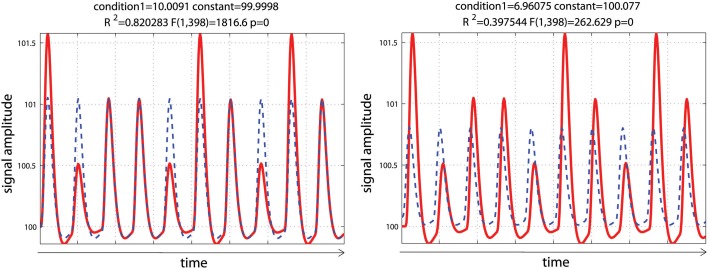
**Periodic event related designs with 1 condition**. In the 1st model **(left)**, the hemodynamic response always start and peak at the same time after stimulus onset (as described in the design matrix) such as the model (blue dashed lines) reflects well the data (red lines). In the 2nd model **(right)**, the hemodynamic response is delayed by 2 s, leading to a poorer fit of the model, reducing *R*^2^/*F*/*p*-values.

Using a set of functions [here the hemodynamic response function (hrf) and its time derivative] rather than the hrf alone is usually considered desirable, because even minor miss-specification of the hemodynamic model can result in substantial bias and loss of power, possibly inflating the type I error rate (Lindquist et al., 2009). In this simulation, data mimicked a periodic event related design with one condition presented at 0.05 Hz. Data corresponded to 10 events of various intensities to reflect some variations in the signal, convolved using a standard hemodynamic response function (i.e., a double gamma function—Friston et al., 1998) with a time resolution of 0.5 s. To demonstrate the impact of adding basis functions on parameter estimates, events were modeled with or without temporal shift (+2 s) relative to the design matrix. As expected, miss-specification of the hemodynamic timing led to a decrease of the parameter estimate and a decrease in model fit (*R*^2^—Figure 4).

**Figure 5:**
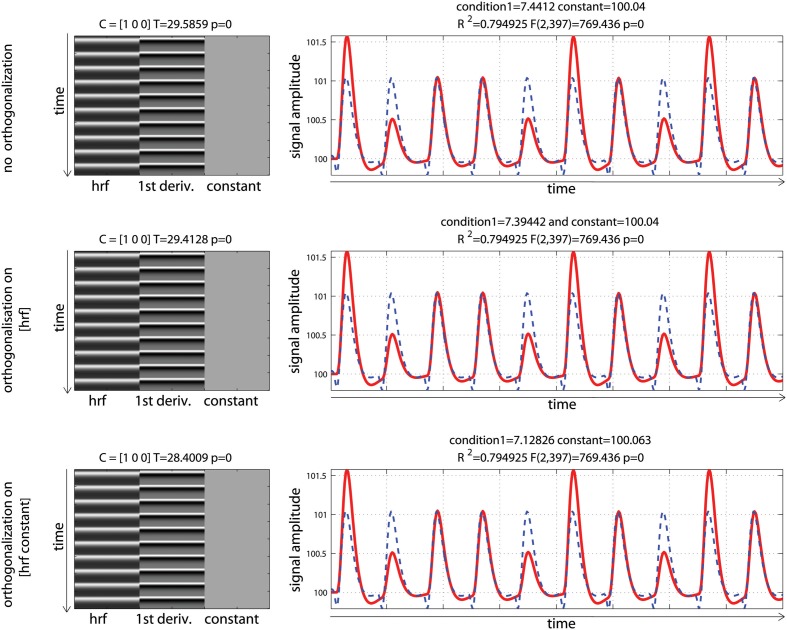
**Periodic event related designs with 1 condition with a temporal shift of 2 s between the model and the data**. From **top** to **bottom**, data are modeled using the hrf and 1st derivative without orthogonalization, using the hrf and 1st derivative with orthogonalization on the hrf only, and the hrf and 1st derivative with orthogonalization on the hrf and constant. Adding derivatives improved the model fit (see *R*^2^, Figure 3) and all 3 designs give the same result. Because of the way regressors are orthogonalized, the weight attributed to the hrf alone however varies, also affecting the *T*-value when tested against 0.

Data were analyzed using design matrices where events were convolved by the hrf (model 1) vs. the hrf and its 1st derivative. Three models were compared: adding the derivative without orthogonalization (model 2), adding the derivative orthogonalized onto the regressor convolved by the hrf [SPM (Friston et al., 2007)—http://www.fil.ion.ucl.ac.uk/spm/] and AFNI styles [(Cox, 1996—http://afni.nimh.nih.gov/afni), model 3], and adding the derivative orthogonalized against the rest of the design matrix [i.e., the regressor convolved by the hrf and the constant term; FSL style, Jenkinson et al. (2012)—http://fsl.fmrib.ox.ac.uk/fsl/, model 4]. Results showed that adding the 1st derivative improved the overall model fit, giving a higher *R*^2^, which is expected since more variance was explained compared with the hrf alone model (Figure 5). Of particular interest here is the behavior of the parameter estimates. In the simulation presented here, the incorrect model gives β_1_ = 6.96 (vs. 10 expected) and adding the temporal derivative, irrespective of orthogonalization, led to an increase of the hrf parameter estimates (7.44 for model 2, 7.39 for model 3, and 7.12 for model 4) thus giving a better estimate of the true hrf regressor. However, when applying the same simulation with the hemodynamic signal peaking earlier than the standard hrf model, adding the temporal derivative has the opposite effect, i.e., it gives lower estimates of the true hrf regressor (see annex 3). Because the true response is however not known with real data, one wants to minimize this effect whilst still accounting for time or dispersion miss-specification. This can be achieved, in theory, by orthogonalizing the derivative(s) regressors with regard to the regressor convolved by the hrf. Orthogonalization also has the advantage to make clear the relative contribution of each regressor to the model (Andrade et al., 1999). Once orthogonalized, the maximum variance is attributed to the regressor convolved by the hrf and additional variance is explained by the orthogonalized regressor convolved by the hrf 1st derivative. In the simulation presented here, this was the 4th model (orthogonalization against the rest of design matrix) which was the most accurate followed by model 3 (orthogonalization against the regressor convolved by the hrf) and finally model 2 (no orthogonalization).

Another important point to notice, it that despite orthogonalization, the parameter estimate for the regressor convolved by the hrf was different before and after adding the temporal derivative. It must be understood that this change in parameter estimate is function of (1) how the orthogonalization is performed (as exemplified above); (2) the correlation between the regressor of interest and the constant term (which itself depends on the inter-stimulus interval—see annex 4); and (3) the presence of other regressors and their degree of correlation. This issue must not be disregarded because results can change drastically between different models (adding derivatives or not, orthogonalization method) and designs (inter-stimulus interval and correlation between regressors).

## Computing the percentage signal change

Rather than using the raw parameter estimates to report or investigate local changes, it is often preferable to compute a more standard measure such as the Percentage Signal Change (PSC). The PSC is defined here as the ratio between the magnitude of the BOLD response and the overall mean of the adjusted time series. Because the parameter estimates from the GLM (Equation 2) are a scaled version of this magnitude, it is also mandatory to account for the value range in the design matrix (Poldrack et al., 2011). The PSC is thus computed as

(7) $$\text{PSC}={\overset{⌢}{\beta }}_{\text{condition}}*SF/{\overset{⌢}{\beta }}_{\text{constant}}*100$$

(8) $$SF=max\text{​}\left(TrialX_{ss}\right)$$

*with* $\overset{⌢}{\beta }$_condition_ *the parameter estimates for a condition of interest*, $\overset{⌢}{\beta }$_constant_ *the parameter estimates for the constant term, SF the scale factor corresponding to the maximum value of a reference trial computed at the resolution of the super-sampled design matrix X_ss_*.

As explained below, the SF not only allows recovering the true signal change but also allows comparing results across different designs. Therefore, instead of using the maximum of a given trial in the experimental design matrix, we may choose a “typical” trial which does not have to be present in the actual design (Poldrack et al., 2011), by default a single event convolved by the super-sampled double gamma-function.

To evaluate the impact of hemodynamic modeling on the computation of PSC and *T*/*p*-values, two event related designs were simulated: the same periodic event related designs as in the previous section (i.e., one experimental event presented at 0.05 Hz) and a randomized event related design for which the experimental condition can occur at closer time interval. In both cases, data corresponded to 10 events with identical mean signals over time. For the first set of analyses, design matrices were created by indicating the onset of each event and convolving the regressor with the double gamma function, with a time resolution of 0.5 s. For the second set of analyses, the data and the design matrices were down-sampled (without interpolation) to correspond to data acquired with a TR of 2 s. This 2nd analysis is crucial because in most software, the design matrix is constructed using a super-sampled hrf (referred to as *X_ss_* Equation 8) and then down-sampled at the resolution of the TR (leading to the design matrix *X*, Equation 1). In each case, the PSC as well as a *t*-test for the effect of the regressor of interest were computed.

**Figure 6:**
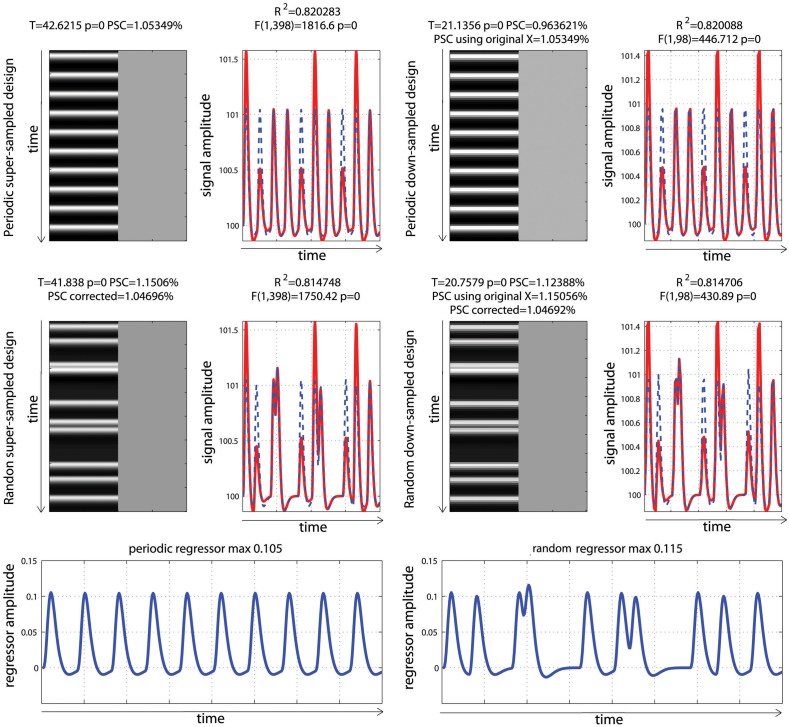
**Periodic vs. random event related designs with a single “active” condition**. On the left are displayed the original design matrices and data and on the right their down-sampled version. PSC stands for Percentage Signal Change computed using the parameters from the hrf and a scaling factor taken as the maximum of the design matrix. PSC using original X (for down sampled data only), stands for Percentage Signal Change computed using the parameters from the hrf and a scaling factor taken as the maximum of the super-sampled design matrix. PSC corrected stands for Percentage Signal Change computed using the parameters from the hrf and a scaling factor taken as the maximum of a reference trial in the super-sampled design matrix.

For both the periodic design and the fast event related designs, the data were created so that the mean activity was identical; with GLM parameters being different (Figure 6). As illustrated, the convolution of the regressor of interest gave identical hrf for each stimulus in the periodic design because stimuli were sufficiently spaced in time. In contrast, the convolution of the regressor of interest in the random design gave different shapes and heights across trials, because stimuli could be closer in time, and convoluted responses accumulated, which reflects real physiological responses. As a consequence of having different shapes and heights in this second design, and since data fitting consists in minimizing the distance between the model and the data, a small (negligible) decrease in total variance explained was observed. This is not to say that random event related designs should not be used: on the contrary, having highly variable designs is more desirable overall (Friston et al., 1999) by maximizing variance between conditions.

### Computing PSC

To be comparable between designs, computation of the PSC has to account for differences in the height of the regressors because, as illustrated in Figure 3, parameter estimate values depend on the scaling of the design matrix. Yet, in none of the studies surveyed (Figure 1) a scaling factor was reported, or maybe even computed. In the examples of Figure 6, the PSC is of ~1.05, and if one computes the PSC without accounting for the scaling factor, we obtain 10 and 9.9%. The PSC is wrong by a factor of 10 (with real data this is unlikely to observe such a large error but it shows here how important this is to account for the design height). However because in both cases the same scaling factor was used (i.e., not using any is like using 1), the difference between the two PSC estimates is small (=0.0620%). This means that PSC reported are often wrong in absolute terms but, assuming that 2 studies have similar designs, the reported values could be roughly compared. Whilst, no studies that computed the PSC manually reported a scaling factor or accounted for the design matrix height, ~12% of studies that reported a PSC were likely to report the right absolute PSC by using toolboxes such as MarsBaR (Brett et al., 2002) which does account the height of regressors by computing the PSC using the fitted response using a reference trial. However, in those cases it is also mandatory to report which parameters were used in those toolboxes as they often offers several options.

In the simulations presented here, the maximum height in the periodic design was 0.105 vs. a maximum height in the fast event related design of 0.115. If one scales the PSC using those heights, we obtained values of 1.05 vs. 1.15%, even though the true signal changes are comparable. In contrast, if one uses the same SF for both designs, the estimates PSC become comparable. If one uses a SF of 0.105, we obtained 1.05% for the period design vs. 1.04% for the fast event related design. If one uses a SF 0.115, the PSC of the periodic design goes up to 1.157 vs. 1.1506% for the fast event related design. This simply illustrates that the PSC is a relative metric. To use the same analogy as Poldrack et al. (2011), the reference trial used to obtain the SF can be thought at as a currency. Looking at the stock market, we can compute the PSC of shares in US dollars or in British pounds. In both cases we use PSC but the absolute values will differ. If the currency is known, we can however convert the PSC from one currency to the other. Similarly if the SF is reported, the can convert the PSC observed in one design to the PSC observed in another design. As proposed, one can use a single trial height computed at the resolution of the super-sampled design matrix as the default currency. In that case we obtain 1.05% for the period design vs. 1.04% for the fast event related design (annex 5).

### Accounting for model sampling

Analysis of the down-sampled data showed that the models before/after down-sampling explained about the same amount of variance as with the original data. *F*-values for the model and *T*-values for the effect of interest were however different because of the difference in the number of observations (degrees of freedom of the error). More importantly, the PSC for down-sampled models were biased and could only be obtained by using a scaling factor from a trial computed at the resolution of the super-sampled design matrix. The reason for this effect is that the minimum or maximum of the hrf can be missed in the down-sampled designs. This an important aspect related to the SF, and users of fMRI software must re-compute either the super sampled design matrix or obtain the reference trial at that resolution (see annex 5 for details as well as specific code for SPM users).

### PSC accounting for time shift

To finish this tutorial, we considered how timing misspecification also impacts PSC computations. Analyses of the high resolution designs were replicated but using data with a temporal shift of +2 s, and a design matrix including the hrf and its time derivative. For these analyses, we compared the PSC computed using the parameter estimates of the hrf to the corrected parameter estimates, which are based on the combination of the hrf and its derivative (Steffener et al., 2010—Equation 9). This correction is required as the magnitude of the hrf is biased because of the temporal shift (Calhoun et al., 2004).

(9) $$H=\sqrt{{\overset{⌢}{\beta }}_{1}^{2}\sum_{1}^{N}x1^{2}+{\overset{⌢}{\beta }}_{2}^{2}\sum_{1}^{N}x2^{2}}*\frac{{\overset{⌢}{\beta }}_{1}}{\left|{\overset{⌢}{\beta }}_{1}\right|}$$

*with H the combined parameter (i.e., amplitude of the hrf accounting for the shift of the derivative), $\overset{⌢}{\beta }$_1_ the parameter estimates for the hrf, *x*_1_ the regressor convolved by the hrf, $\overset{⌢}{\beta }$_2_ the parameter estimates for the temporal derivative, *x*_2_ the regressor convolved by the 1st derivative*. Note the difference with Steffener et al. (2010), here there is a post-multiplication of $\overset{⌢}{\beta }$_1_ divided by its absolute value, allowing recovering the sign [as in Calhoun et al. (2004)].

**Figure 7:**
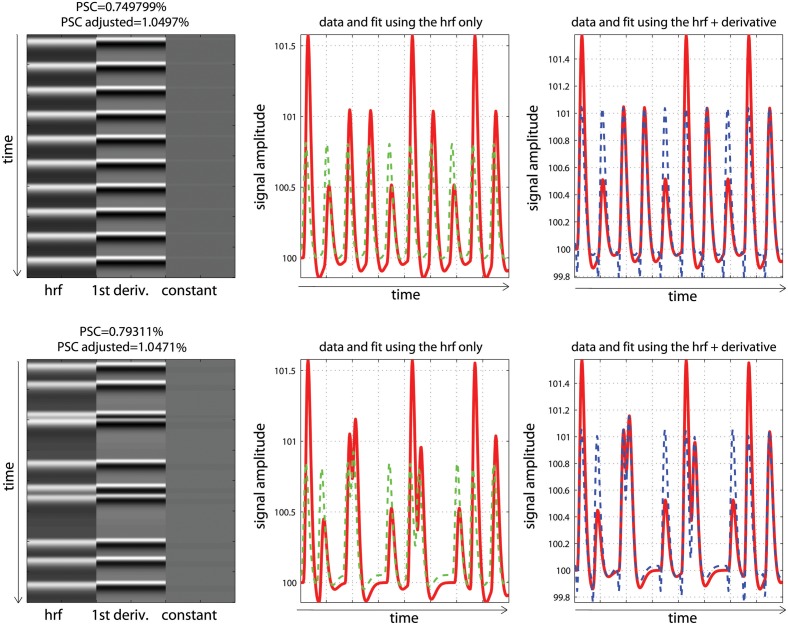
**Periodic (top) vs. random (bottom) event related designs with a temporal shift in the data**. The fitted data using the hrf only (green) are misaligned with the signal (red), leading to under-estimate the magnitude of the response and thus the PSC. The magnitude is better estimated using the hrf and derivative (blue) giving more accurate PSC.

Analysis and modeling of the periodic vs. fast event related designs with some temporal delay are displayed in Figure 7. As illustrated, fitted data using the hrf regressors only were misaligned with the observed responses, leading to smaller parameter estimates than expected. Consequently, computing the PSC using these parameters also gave smaller values: 0.74 and 0.79%. In contrast, adding temporal derivatives improved the data fit, and fitted data were aligned with the signal. Using the height of the fitted data (i.e., the combination of parameters from the hrf and derivatives—Equation 9) therefore returned much closer estimates of the PSC (here 1.03 and 1.07%).

## Discussion

The first misconception about the GLM has to do with modeling rest or null events and can be related to the understanding of (1) what the constant term is (2) what model (over) parameterization implies. Because the constant term is often referred to as the intercept, this is often interpreted as “baseline.” Physically, the constant term reflects the offset of the measured signal, which is not on average zero even without stimuli (Poline et al., 2007). Mathematically, it corresponds to the intercept of the full design matrix in n dimensions, meaning it is the estimated average of the data adjusted for all of the other effects in the design matrix (the adjusted mean). For instance, in a simple regression, the adjusted data are the data minus a single (linear trend) effect and the constant term is the mean of these adjusted data (i.e., the intercept of the regression line). In a controlled block design, the adjusted data are the data minus the modeled activation blocks and the constant is the mean of this adjusted data (i.e., it reflects rest periods). Intuitively, it therefore makes sense to not model fMRI “rest” periods or null events. At a more fundamental level, modeling baseline leads to rank deficient, i.e., non-invertible, matrices which implies that the model parameters are not estimable, i.e., that nothing can be said about their individual value. In practice, after convolution of the regressors by the hrf model, the design matrix is most often not rank deficient, but is close to being singular which leads to poor/unstable estimates as well. In addition, in fMRI, the actual parameters and noise estimates are also affected by the noise model [white, AR(1)…] added to Equation 1 (see e.g., Monti, 2011).

Modeling rest periods or null events does not, however, necessarily impact statistical results (second misconception), as long as the right contrasts are used. For a simple block design (Figure 2) modeling both “activation” and “rest” gave the wrong estimates compared to the true underlying signal change, and the contrast [0 1 0] returned the wrong *T*-value for testing activations alone, while a contrast [–1 1 0] testing activation vs. rest was valid, since the difference between activation and rest was the same as the one obtained from parameter estimates of a well-parameterized model. The same applies for any other designs (see e.g., Figure 2). In terms of group analysis, this means that for models that do include rest or null events, the group statistics is valid as long as it is based on a contrast between conditions. Any use of individual parameter estimates (which is the same as using a contrast like e.g., [1 0]) for modeling at the 2nd level but also for plotting effect sizes (beta or PSC) or performing ROI analyses, is however invalid and again only contrast values must be used. This last point is not trivial. Most fMRI studies do not report enough information about effect sizes to allow comparisons between studies and prospective power computations. It is essential that more plots and parameter estimates (or PSC) of the observed effects are reported, but no need to say that these reported values need to be valid.

A third misconception is to think that adding temporal and/or dispersion derivatives never change the parameter estimate(s) of the hrf regressor(s), because of orthogonalization. In a linear system, fitting orthogonal regressors is indeed identical as fitting each regressor separately because orthogonal regressors are also uncorrelated (for further insight into independence vs. orthogonality vs. correlation, see Rodgers et al., 1984). In the GLM as used in fMRI, regressors are however never all uncorrelated. Even for the simple single event related design as in Figure 3, the regressor of the event is correlated with the constant to some degree. Therefore, if one adds the orthogonalized 1st derivative, and depending on the software orthogonalization procedure, the parameter estimate of the hrf regressor can change (see also annex 4). It is thus essential for users to know exactly how orthogonalization is performed in the software they use. In most cases, parameters will change because there is also more than one condition and the correlations between regressors across different conditions are likely to change after orthogonalization. Including derivatives at the 1st level therefore also impact on group results which only include the regressors from the hrf. In most cases it is recommended to perform the analyses twice (with and without derivatives) and examine differences carefully. Alternatively, combined estimates may be used at the second level (Calhoun et al., 2004) alleviating these issues.

The final and forth misconception related to the PSC. First, it is essential to define relative to what the PSC is computed. If using the GLM parameter estimates, the PSC is computed relative to the adjusted mean (which can be seen as the baseline in block designs or event related designs). In other cases, like the default in AFNI, this is relative to the temporal mean. This has to be reported because the actual values will differ between methods, even for the same data. Second, when using the GLM parameter estimates, it is also essential to define a reference trial at the resolution of the super-sampled design and report the scaling factor because of (1) the impact of the design matrix data range (scaling) on parameter estimates, (2) the impact of data resolution (i.e., TR) compared to the hemodynamic model, and (3) the differences in the way hemodynamic responses can summate. Unfortunately most software do not provide such information easily and one needs to regenerate the super-sampled model or recreate a “typical” trial. For group analyses, if the 2nd level was performed using the parameters of regressors convolved by hrf only, it makes sense to report the PSC computed using these same parameters (and use a scaling factor based on a reference trial sampled according to the design). If the analysis, however, uses derivatives and/or there are evidences of a temporal shift or narrowing/widening of the hfr, using a combination of parameters is more likely to reflect the true PSC as demonstrated in the simulation.

### Conflict of interest statement

The author declares that the research was conducted in the absence of any commercial or financial relationships that could be construed as a potential conflict of interest.

## Annex 1

The abstract of all articles published in the journal Neuroimage between January 2013 and June 2013 were examined, following which studies that used fMRI were retained (human and non-human alike). The method part of each of these articles was examined and only studies using the “standard” GLM procedure were included in the review, i.e., studies using in house methods, block averaging, FIR models or using multivariate pattern analyses were discarded. In total, 75 articles were included and listed below in an abbreviated reference format.

For each article, the following methodological criteria were recorded:

(i) Blocked or event related design,
(ii) Rest periods or null events presents,
(iii) Modeling of rest or null events if any,
(iv) Reporting of effect size estimates (beta/con or PSC),
(v) Were reported parameter estimates valid (i.e. using a contrast if rest was modeled),
(vi) If PSC reported, how was it computed.

Articles included in the review:

Agnew et al. (2013). *Neuroimage* 73, 191–199.

Andics et al. (2013). *Neuroimage* 69, 277–283.

Apps et al. (2013). *Neuroimage* 64, 1–9.

Archila-Suerte et al. (2013). *Neuroimage* 51–63.

Baeck et al. (2013). *Neuroimage* 70, 37–47.

Bennett et al. (2013). *Neuroimage* 72, 20–32.

Bergstrom et al. (2013). *Neuroimage* 58, 141–153.

Blank and von Kriegstein (2013). *Neuroimage* 65, 109–118.

Bonath et al. (2013). *Neuroimage* 66, 110–118.

Bonner et al. (2013). *Neuroimage* 71, 175–186.

Bonzano et al. (2013). *Neuroimage* 65, 257–266.

Bradley et al. (2013). *Neuroimage* 67, 101–110.

Brown et al. (2013). *Neuroimage* 54, 458–465.

Callan et al. (2013). *Neuroimage* 72, 55–68.

Callan et al. (2013). *Neuroimage* 66, 22–27.

Campanella et al. (2013). *Neuroimage* 71, 92–103.

Causse et al. (2013). *Neuroimage* 71, 19–29.

Chen et al. (2013). *Neuroimage* 66, 577–584.

Cohen Kadosh et al. (2013). *Neuroimage* 69, 11–20.

Cservenka et al. (2013). *Neuroimage* 66, 184–193.

de Hass et al. (2013). *Neuroimage* 70, 258–267.

Demanet et al. (2013). *Neuroimage* 72, 207–213.

Di Dio et al. (2013). *Neuroimage* 54, 425–436.

Egidi and Caramazza (2013). *Neuroimage* 71, 59–74.

Ferri et al. (2013). *Neuroimage* 70, 268–277.

Fischer et al. (2013). *Neuroimage* 66, 261–269.

Freud et al. (2013). *Neuroimage* 64, 685–692.

Gilead et al. (2013). *Neuroimage* 65, 267–279.

Gillebert et al. (2013). *Neuroimage* 67, 257–272.

Gorgolewski et al. (2013). *Neuroimage* 69, 231–243.

Heinzel et al. (2013). *Neuroimage* 71, 125–134.

Helbing et al. (2013). *Neuroimage* 64, 43–60.

Henderson and Norris (2013). *Neuroimage* 64, 582–589.

Hermans et al. (2013). *Neuroimage* 66, 278–287.

Hove et al. (2013). *Neuroimage* 67, 313–321.

Indovina et al. (2013). *Neuroimage* 71, 114–124.

James and James (2013). *Neuroimage* 67, 182–192.

Kassuba et al. (2013). *Neuroimage* 65, 59–68.

Kau et al. (2013). *Neuroimage* 64, 299–307.

Killgore et al. (2013). *Neuroimage* 71, 216–223.

Kitayama et al. (2013). *Neuroimage* 69, 206–212.

Koritzky et al. (2013). *Neuroimage* 72, 280–286.

Kruger et al. (2013). *Neuroimage* 64, 197–208.

Kulakova et al. (2013). *Neuroimage* 72, 265–271.

Liang et al. (2013). *Neuroimage* 64, 104–111.

Liew et al. (2013). *Neuroimage* 69, 138–145.

Limongi et al. (2013). *Neuroimage* 71, 147–157.

Lutz et al. (2013). *Neuroimage* 54, 538–546.

Lutz et al. (2013). *Neuroimage* 66, 288–292.

Madlon-Kay et al. (2013). *Neuroimage* 70, 66–79.

Manginelli et al. (2013). *Nuroimage* 67, 363–374.

Moon et al. (2013). *Neuroimage* 64, 91–103.

Muller et al. (2013). *Neuroimage* 66, 361–367.

Pau et al. (2013). *Neuroimage* 64, 379–387.

Pomares et al. (2013). *Neuroimage* 54, 466–475.

Raabe et al. (2013). *Neuroimage* 71, 84–91.

Rothermich and Kotz (2013). *Neuroimage* 70, 89–100.

Simonyan et al. (2013). *Neuroimage* 70, 21–32.

Spreckelmeyer et al. (2013). *Nuroimage* 66, 223–231.

Stice et al. (2013). *Neuroimage* 67, 322–330.

Sun et al. (2013). *Neuroimage* 65, 23–33.

Sutherland et al. (2013). *Neuroimage* 66, 585–593.

Telzer et al. (2013). *Neuroimage* 71, 275–283.

Thornton and Conway (2013). *Neuroimage* 70, 233–239.

Turk-Browne et al. (2013). *Neuroimage* 66, 553–562.

Tusche et al. (2013). *Neuroimage* 72, 174–182.

Tyll et al. (2013). *Neuroimage* 65, 13–22.

van der Heiden et al. (2013). *Neuroimage* 65, 387–394.

van der Zwaag et al. (2013). *Neuroimage* 67, 354–362.

Witt and Stevens (2013). *Neuroimage* 65, 139–151.

Wu et al. (2013). *Neuroimage* 65, 466–475.

Yu-Chen et al. (2013). *Neuroimage* 66, 169–176.

Zeki and Stutters (2013). *Neuroimage* 73, 156–166.

Zhang and Hirch (2013). *Neuroimage* 65, 223–230.

Zhang et al. (2013). *Neuroimage* 65, 119–126.
