# Supplementary material for: Misconceptions in the use of the General Linear Model applied to functional MRI: a tutorial for junior neuro-imagers
Source: Front Neurosci. 2014 Jan 21;8:1. doi: 10.3389/fnins.2014.00001 (PMC3896880; doi:10.3389/fnins.2014.00001)
Supplement: Supplementary file 1 [file DataSheet1.ZIP › Annex 2.pdf]

Annex 2

Model parameterization

This Matlab code is a simple demonstration of how to code design matrices for fMRI. For simplicity, no convolution is involved (i.e. no model of the haemodynamic response function). The principles developed however do apply to fMRI analyses.

Controlled block design

First create the data alternating the control condition (baseline) set at 10, with the experimental condition set at 11, i.e. 10% change condition > baseline

```
clear
baseline = [9.9 10 10.1];     % average 10
condition = [10.9 11 11.1];   % average 11
Y = [baseline condition baseline condition baseline condition]';
SStotal = norm(Y-mean(Y)).^2; % this is the total sum of squares
```

No matter the design matrix, each time we can solve the equation $Y = X * \beta + e$

by computing $\hat{\beta} = X^{+} * Y$

The fitted data are thus $\hat{Y} = X * \hat{\beta}$

The residuals are thus $Res = Y - \hat{Y}$

Consider a model where all events are modelled

```
X1 = [repmat([kron(eye(2),ones(3,1))],3,1) ones(18,1)];
beta1 = pinv(X1)*Y;
Yhat = X1*beta1;
Residuals = Y - Yhat;

% the model statistics are
SSeffect = norm(Yhat-mean(Yhat)).^2;           % sum of squares of the effect
SSerror = norm(Residuals-mean(Residuals)).^2; % sum of squares of the error
R2 = SSeffect / SStotal;                       % R square value (amount of variance explained)
df = rank(X1)-1;                               % degrees of freedom of the model
dfe = length(Y)-rank(X1);                      % degrees of freedom of the error
F = (SSeffect/df) / (SSerror/dfe);             % F value
p_val = 1-spm_Fcdf(F,df,dfe);                  % p value
```

The statistics for a given condition can be obtained using a contrast. For a details on computations performed below see e.g. Human Brain Function book
http://www.fil.ion.ucl.ac.uk/spm/doc/books/hbf2/pdfs/Ch7.pdf

```
figure('Name','Fig. 2 Controlled block design');
subplot(3,2,1); imagesc(X1);colormap('gray');
subplot(3,2,2); stairs([1:19],[Y;10],'r','LineWidth',3); axis([1 20 9.8 11.2])
hold on; stairs([1:19],[Yhat;11],'--','LineWidth',3); grid on;
mytitle = sprintf('baseline=%g condition=%g constant=%g \n R^2=%g F(%g,%g)=%g p=%g',
```

```
beta1(1), beta1(2), beta1(3), R2, df, dfe, F, p_val);
title(mytitle,'FontSize',14)

P = X1*pinv(X1);                                     % Projection matrix onto X
R = eye(size(Y,1)) - P;                              % Projection onto null space
variance = ((R*Y)'*(R*Y)) / (size(Y,1)-rank(X1));
C = [0 1 0];                                         % contrast for condition
T_con = (C*beta1) ./ sqrt(variance.*(C*pinv(X1'*X1)*C')); % T value
p_con = 2*(1-spm_Tcdf(T_con, (size(Y,1)-rank(X1))));
fprintf('Over parameterized model: activation versus 0 T=%g p=%g \n',T_con,p_con)

% update figure
mytitle = sprintf('Over parameterized design matrix \n C=[0 1 0] T=%g p=%g', T_con, p_con);
subplot(3,2,1); title(mytitle,'FontSize',14)

% However, when explicitely modelling baseline, one usually
% contrast activation versus rest
C = [-1 1 0]; % contrast for condition versus baseline
T_con = (C*beta1) ./ sqrt(variance.*(C*pinv(X1'*X1)*C'));
p_con = 2*(1-spm_Tcdf(T_con, (size(Y,1)-rank(X1))));
fprintf('Over parameterized model: activation versus beaeline T=%g p=%g \n',T_con,p_con)
```

Consider a model where baseline is not modelled

```
X2 = X1; X2(:,1) = [];
beta2 = pinv(X2)*Y;
Yhat = X2*beta2;
Residuals = Y - Yhat;
subplot(3,2,3); imagesc(X2);colormap('gray');
subplot(3,2,4); stairs([1:19],[Y;10],'r','LineWidth',3); axis([1 20 9.8 11.2])
hold on; stairs([1:19],[Yhat;11],'--','LineWidth',3); grid on;

% stats 1
SSeffect = norm(Yhat-mean(Yhat)).^2;
SSerror = norm(Residuals-mean(Residuals)).^2;
R2 = SSeffect / SStotal;
df = rank(X2)-1;
dfe = length(Y)-rank(X2);
F = (SSeffect/df) / (SSerror/dfe);
p_val = 1-spm_Fcdf(F,df,dfe);
mytitle = sprintf('condition=%g constant=%g \n R^2=%g F(%g,%g)=%g p=%g', beta2(1), beta2(2),
R2, df, dfe, F, p_val);
title(mytitle,'FontSize',14)

% stats 2
P = X2*pinv(X2); % H matrix
R = eye(size(Y,1)) - P;
variance = ((R*Y)'*(R*Y)) / (size(Y,1)-rank(X2));
C = [1 0]; % contrast for condition
T_con = (C*beta2) ./ sqrt(variance.*(C*pinv(X2'*X2)*C')); % T value
p_con = 2*(1-spm_Tcdf(T_con, (size(Y,1)-rank(X2))));
mytitle = sprintf('Well parameterized design matrix \n C = [1 0] T=%g p=%g', T_con, p_con);
subplot(3,2,3); title(mytitle,'FontSize',14)
fprintf('Well parameterized model: activation versus implicit baseline T=%g p=%g
\n',T_con,p_con)
```

Consider another model where the design matrix is between 0 and 2

```
X3 = X2; X3(find(X2(:,1)),1) = 2;
beta3 = pinv(X3)*Y;
Yhat = X3*beta3;
Residuals = Y - Yhat;

subplot(3,2,5); imagesc(X3);colormap('gray');
subplot(3,2,6); stairs([1:19],[Y;10],'r','LineWidth',3); axis([1 20 9.8 11.2])
hold on; stairs([1:19],[Yhat;11],'--','LineWidth',3); grid on;

% stats 1
SSeffect = norm(Yhat-mean(Yhat)).^2;
SSerror = norm(Residuals-mean(Residuals)).^2;
R2 = SSeffect / SStotal;
df = rank(X3)-1;
dfe = length(Y)-rank(X3);
F = (SSeffect/df) / (SSerror/dfe);
p_val = 1-spm_Fcdf(F,df,dfe);
mytitle = sprintf('condition=%g constant=%g \n R^2=%g F(%g,%g)=%g p=%g', beta3(1), beta3(2),
R2, df, dfe, F, p_val);
title(mytitle,'FontSize',14)

% stats 2
P = X3*pinv(X3); % H matrix
R = eye(size(Y,1)) - P;
variance = ((R*Y)'*(R*Y)) / (size(Y,1)-rank(X3));
C = [1 0]; % contrast for condition
T_con = (C*beta3) ./ sqrt(variance.*(C*pinv(X3'*X3)*C')); % T value
p_con = 2*(1-spm_Tcdf(T_con, (size(Y,1)-rank(X3))));
mytitle = sprintf('Well parameterized design matrix \n C = [1 0] T=%g p=%g', T_con, p_con);
subplot(3,2,5); title(mytitle,'FontSize',14)
fprintf('Well parameterized model, design matrix scaled up: activation versus implicit
baseline T=%g p=%g \n',T_con,p_con)
```

```
Over parameterized model: activation versus 0 T=185.903 p=0
Over parameterized model: activation versus beaeline T=24.4949 p=4.10783e-14
Well parameterized model: activation versus implicit baseline T=24.4949 p=4.10783e-14
Well parameterized model, design matrix scaled up: activation versus implicit baseline
T=24.4949 p=4.10783e-14

See figure 2 in manuscript
```

Alternating block design

Alternate 2 experimental conditions set at 9 and 11, i.e. 10% change relative to baseline 10

```
clear
condition1 = [8.9 9 9.1];
baseline = [9.9 10 10.1];
condition2 = [10.9 11 11.1];
Y = [condition1 baseline condition2 baseline condition1 baseline condition2 baseline
condition1 baseline condition2]';
SStotal = norm(Y-mean(Y)).^2;
```

Consider a model where all events are modelled

```
X1 = [[[kron(eye(3),ones(3,1))] ; [fliplr([kron(eye(2),ones(3,1))]) zeros(6,1)]; ...
    [zeros(6,1) kron(eye(2),ones(3,1))] ; [zeros(3,1) ones(3,1) zeros(3,1)] ; ...
    [kron(eye(3),ones(3,1))]] ones(33,1)];
beta1 = pinv(X1)*Y;
Yhat = X1*beta1;
Residuals = Y - Yhat;

figure('Name','Fig. 3 Alternating block design');
subplot(3,2,1); imagesc(X1);colormap('gray');
subplot(3,2,2); stairs([1:34],[Y;10],'r','LineWidth',3); axis([1 35 8.7 11.3])
yhat1 = X1(:,[1 4])*beta1([1 4]); hold on; stairs([1:33],[yhat1],'--','LineWidth',3);
yhat2 = X1(:,[2 4])*beta1([2 4]); stairs([1:33],[yhat2],'k--','LineWidth',3); grid on;
yhat3 = X1(:,[3 4])*beta1([3 4]); stairs([1:34],[yhat3;11],'g--','LineWidth',3);

% stats 1
SSeffect = norm(Yhat-mean(Yhat)).^2;
SSerror = norm(Residuals-mean(Residuals)).^2;
R2 = SSeffect / SStotal;
df = rank(X1)-1;
dfe = length(Y)-rank(X1);
F = (SSeffect/df) / (SSerror/dfe);
p_val = 1-spm_Fcdf(F,df,dfe);
mytitle = sprintf('baseline=%g condition1=%g condition2=%g constant=%g \n R^2=%g F(%g,%g)=%g
p=%g', beta1(2), beta1(1), beta1(3), beta1(4), R2, df, dfe, F, p_val);
title(mytitle,'FontSize',14)

% stats 2
P = X1*pinv(X1); % H matrix
R = eye(size(Y,1)) - P;
variance = ((R*Y)'*(R*Y)) / (size(Y,1)-rank(X1));
C = [-1 0 1 0]; % contrast for condition 2 > condition 1
T_con = (C*beta1) ./ sqrt(variance.*(C*pinv(X1'*X1)*C'));
p_con = 2*(1-spm_Tcdf(T_con, (size(Y,1)-rank(X1))));
mytitle = sprintf('Over parameterized design matrix \n C = [-1 0 1 0] T=%g p=%g', T_con,
p_con);
subplot(3,2,1); title(mytitle,'FontSize',14)
fprintf('Over parameterized model: cond2 > cond1 T=%g p=%g \n',T_con,p_con)
```

Consider a model where baseline is not modelled

```
X2 = X1; X2(:,2) = [];
beta2 = pinv(X2)*Y;
Yhat = X2*beta2;
Residuals = Y - Yhat;

subplot(3,2,3); imagesc(X2);colormap('gray');
subplot(3,2,4); stairs([1:34],[Y;10],'r','LineWidth',3); axis([1 35 8.7 11.3])
yhat1 = X2(:,[1 3])*beta2([1 3]); hold on; stairs([1:34],[yhat1;10],'--','LineWidth',3);
yhat2 = X2(:,[2 3])*beta2([2 3]); stairs([1:34],[yhat2;11],'g--','LineWidth',3); grid on

% stats 1
SSeffect = norm(Yhat-mean(Yhat)).^2;
SSerror = norm(Residuals-mean(Residuals)).^2;
R2 = SSeffect / SStotal;
df = rank(X1)-1;
dfe = length(Y)-rank(X1);
F = (SSeffect/df) / (SSerror/dfe);
p_val = 1-spm_Fcdf(F,df,dfe);
mytitle = sprintf('condition1=%g condition2=%g constant=%g \n R^2=%g F(%g,%g)=%g p=%g',
beta2(1), beta2(2), beta2(3), R2, df, dfe, F, p_val);
title(mytitle,'FontSize',14)

% stats 2
P = X2*pinv(X2); % H matrix
R = eye(size(Y,1)) - P;
variance = ((R*Y)'*(R*Y)) / (size(Y,1)-rank(X2));
C = [-1 1 0]; % contrast for condition
T_con = (C*beta2) ./ sqrt(variance.*(C*pinv(X2'*X2)*C'));
p_con = 2*(1-spm_Tcdf(T_con, (size(Y,1)-rank(X2))));
mytitle = sprintf('Well parameterized design matrix \n C = [-1 1 0] T=%g p=%g', T_con,
p_con);
subplot(3,2,3); title(mytitle,'FontSize',14)
fprintf('Well parameterized model: cond2 > cond1 T=%g p=%g \n',T_con,p_con)
```

Consider another model where the design matrix is between 0 and 2

```
X3 = X2;
X3(find(X2(:,1)),1) = 2;
X3(find(X2(:,2)),2) = 2;
beta3 = pinv(X3)*Y;
Yhat = X3*beta3;
Residuals = Y - Yhat;

subplot(3,2,5); imagesc(X3);colormap('gray');
subplot(3,2,6); stairs([1:34],[Y;10],'r','LineWidth',3); axis([1 35 8.7 11.3])
yhat1 = X3(:,[1 3])*beta3([1 3]); hold on; stairs([1:34],[yhat1;10],'--','LineWidth',3);
yhat2 = X3(:,[2 3])*beta3([2 3]); stairs([1:34],[yhat2;11],'g--','LineWidth',3); grid on

% stats 1
SSeffect = norm(Yhat-mean(Yhat)).^2;
SSerror = norm(Residuals-mean(Residuals)).^2;
```

```
R2 = SSeffect / SStotal;
df = rank(X3)-1;
dfe = length(Y)-rank(X3);
F = (SSeffect/df) / (SSerror/dfe);
p_val = 1-spm_Fcdf(F,df,dfe);
mytitle = sprintf('condition1=%g condition2=%g constant=%g \n R^2=%g F(%g,%g)=%g p=%g',
beta3(1), beta3(2), beta3(3), R2, df, dfe, F, p_val);
title(mytitle,'FontSize',14)

% stats 2
P = X3*pinv(X3); % H matrix
R = eye(size(Y,1)) - P;
variance = ((R*Y)'*(R*Y)) / (size(Y,1)-rank(X3));
C = [-1 1 0]; % contrast for condition
T_con = (C*beta3) ./ sqrt(variance.*(C*pinv(X3'*X3)*C'));
p_con = 2*(1-spm_Tcdf(T_con, (size(Y,1)-rank(X3))));
mytitle = sprintf('Well parameterized design matrix \n C = [-1 1 0] T=%g p=%g', T_con,
p_con);
subplot(3,2,5); title(mytitle,'FontSize',14)
fprintf('Well parameterized model, design matrix scaled up: cond2 > cond1 T=%g p=%g
\n',T_con,p_con)
```

```
Over parameterized model: cond2 > cond1 T=49.5434 p=0
Well parameterized model: cond2 > cond1 T=49.5434 p=0
Well parameterized model, design matrix scaled up: cond2 > cond1 T=49.5434 p=0
```

See figure 3 in manuscript
