# Supplementary material for: Misconceptions in the use of the General Linear Model applied to functional MRI: a tutorial for junior neuro-imagers
Source: Front Neurosci. 2014 Jan 21;8:1. doi: 10.3389/fnins.2014.00001 (PMC3896880; doi:10.3389/fnins.2014.00001)
Supplement: Supplementary file 1 [file DataSheet1.ZIP › Annex 3.pdf]

Annex 3

Derivative effect

Simulation of event related designs analyzed using hrf+derivatives to show the effect of adding derivatives and also how parameter estimate can change depending on the procedure. The code calls spm_get_bf.m which is the SPM http://www.fil.ion.ucl.ac.uk/spm/ function to obtain the hrf and derivatives. It also calls spm_orth.m which is the orthogonalization procedure used in SPM. Finally, it calls spm_orth2.m (available to download with the tutorial paper) which is a modification of spm_orth.m such as derivatives are orthogonalized against the hrf regressor and constant term together.

```
clear all
shift = 2; % enter here the delay in seconds
           % e.g. 2 means the data peak later than expected
           % -2 means the data peak earlier than expected


% hrf model using SPM function
% ---------------------------
xBF.dt = 0.5;
xBF.name = 'hrf (with time derivative)';
xBF.length = 32;
xBF.order = 1;
xBF = spm_get_bf(xBF);

figure('Name','hrf model')
plot(xBF.bf,'LineWidth',3); axis tight; grid on;
```

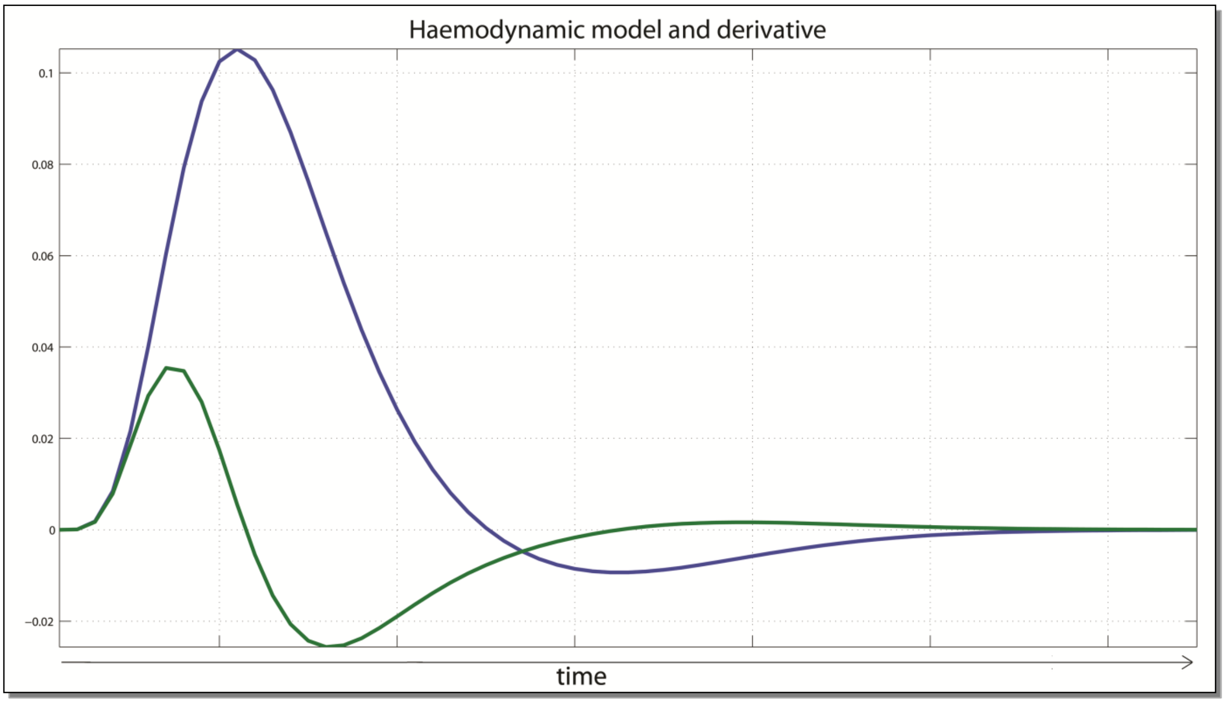

Supplementary Figure 1. Haemodynamic model and 1st derivative obtained from spm_get_bf.m

Compare data with / without temporal shift

No temporal shift

```
scale = [15 5 10 10 5 15 10 5 15 10]; % height of the neural response

onsets = 1:40:400;
Y1 = zeros(500,1); % the data, 250 sec sample at 2Hz
X1 = zeros(500,1); % the regressor of the matrix
for i=1:10
    Y1(onsets(i)) = scale(i);
    X1(onsets(i)) = 1;
end

SS = conv(X1,spm_hrf(0.5));   % super-sampled regressor
X1 = [SS(1:400) ones(400,1)]; % adjust length for simulation
Y1 = conv(Y1,spm_hrf(0.5));   % simulated signal
Y1 = Y1(1:400)+100;           % adjust length for simulation
SStotal = norm(Y1-mean(Y1)).^2;

beta1 = pinv(X1)*Y1;
Yhat = X1*beta1;
figure('Name','Fig. 4 Effect of temporal shift')
subplot(2,4,[1 5]); imagesc([zscore(X1(:,1)) X1(:,2)]);colormap('gray');
subplot(2,4,[3 4]); plot(Y1,'r','LineWidth',3); grid on; axis tight
hold on; plot(Yhat,'--','LineWidth',2); grid on; axis tight

% stats 1
SSeffect = norm(Yhat-mean(Yhat)).^2;
Residuals = Y1 - Yhat;
SSerror = norm(Residuals-mean(Residuals)).^2;
R2 = SSeffect / SStotal;
df = rank(X1)-1;
dfe = length(Y1)-rank(X1);
F = (SSeffect/df) / (SSerror/dfe);
p_val = 1-spm_Fcdf(F,df,dfe);
mytitle = sprintf('condition1=%g constant=%g \n R^2=%g F(%g,%g)=%g p=%g', beta1(1), beta1(2),
R2, df, dfe, F, p_val);
title(mytitle,'FontSize',14)

% stats 2
P = X1*pinv(X1);
R = eye(size(Y1,1)) - P;
variance = ((R*Y1)'*(R*Y1)) / (size(Y1,1)-rank(X1));
C = [1 0];
T_con = (C*beta1) ./ sqrt(variance.*(C*pinv(X1'*X1)*C'));
p_con = 2*(1-spm_Tcdf(T_con, (size(Y1,1)-rank(X1))));
myrange(1) = range(X1(:,1));
mytitle = sprintf('good onsets \n C = [1 0] T=%g p=%g', T_con, p_con);
subplot(2,4,[1 5]); title(mytitle,'FontSize',14)
```

Shift the signal in time

```
Y1 = zeros(500,1); % 250 sec sample at 2Hz
X1 = zeros(500,1);
for i=1:10
    try
        Y1(onsets(i)+shift*2) = scale(i); % shift
    catch
        Y1(onsets(i)) = scale(i); % no shift - occurs for neg value at Y(1)
    end
    X1(onsets(i)) = 1;
end

SS(:,1) = conv(X1,xBF.bf(:,1));
SS(:,2) = conv(X1,xBF.bf(:,2));
X1 = [SS(1:400,1) ones(400,1)];
Y1 = conv(Y1,spm_hrf(0.5));
Y1 = Y1(1:400)+100;
SStotal = norm(Y1-mean(Y1)).^2;

beta1 = pinv(X1)*Y1;
Yhat = X1*beta1;
subplot(2,4,[2 6]); imagesc([zscore(X1(:,1)) X1(:,2)]);colormap('gray');
subplot(2,4,[7 8]); plot(Y1,'r','LineWidth',3); grid on; axis tight
hold on; plot(Yhat,'--','LineWidth',2); grid on; axis tight

% stats 1
SSeffect = norm(Yhat-mean(Yhat)).^2;
Residuals = Y1 - Yhat;
SSerror = norm(Residuals-mean(Residuals)).^2;
R2 = SSeffect / SStotal;
df = rank(X1)-1;
dfe = length(Y1)-rank(X1);
F = (SSeffect/df) / (SSerror/dfe);
p_val = 1-spm_Fcdf(F,df,dfe);
mytitle = sprintf('condition1=%g constant=%g \n R^2=%g F(%g,%g)=%g p=%g', beta1(1), beta1(2),
R2, df, dfe, F, p_val);
title(mytitle,'FontSize',14)

% stats 2
P = X1*pinv(X1); % H matrix
R = eye(size(Y1,1)) - P;
variance = ((R*Y1)'*(R*Y1)) / (size(Y1,1)-rank(X1));
C = [1 0];
T_con = (C*beta1) ./ sqrt(variance.*(C*pinv(X1'*X1)*C'));
p_con = 2*(1-spm_Tcdf(T_con, (size(Y1,1)-rank(X1))));
mytitle = sprintf('bad onsets \n C = [1 0] T=%g p=%g', T_con, p_con);
subplot(2,4,[2 6]); title(mytitle, 'FontSize',14)
```

See figure 4 in manuscript

Model the time shifted signal using the 1st derivative

No orthogonalization

```
X2 = [SS(1:400,:) ones(400,1)];
beta2 = pinv(X2)*Y1;
Yhat = X2*beta2;
figure('Name','Fig. 5. Models with derivatives');
subplot(3,3,1); imagesc([zscore(X2(:,1:2)) X2(:,3)]) ;colormap('gray');
subplot(3,3,[2 3]); plot(Y1,'r','LineWidth',3); grid on; axis tight
hold on; plot(Yhat,'--','LineWidth',2); grid on; axis tight

% stats 1
SSeffect = norm(Yhat-mean(Yhat)).^2;
Residuals = Y1 - Yhat;
SSerror = norm(Residuals-mean(Residuals)).^2;
R2 = SSeffect / SStotal;
df = rank(X2)-1;
dfe = length(Y1)-rank(X2);
F = (SSeffect/df) / (SSerror/dfe);
p_val = 1-spm_Fcdf(F,df,dfe);
mytitle = sprintf('condition1=%g constant=%g \n R^2=%g F(%g,%g)=%g p=%g', beta2(1), beta2(3),
R2, df, dfe, F, p_val);
title(mytitle,'FontSize',14)

% stats 2
P = X2*pinv(X2); % H matrix
R = eye(size(Y1,1)) - P;
variance = ((R*Y1)'*(R*Y1)) / (size(Y1,1)-rank(X2));
C = [1 0 0];
T_con = (C*beta2) ./ sqrt(variance.*(C*pinv(X2'*X2)*C'));
p_con = 2*(1-spm_Tcdf(T_con, (size(Y1,1)-rank(X2))));
mytitle = sprintf('C = [1 0 0] T=%g p=%g', T_con, p_con);
subplot(3,3,1); title(mytitle,'FontSize',14)
```

Derivative orthogonal to hrf regressor

```
x = spm_orth(SS);
X3 = [x(1:400,:) ones(400,1)];
beta3 = pinv(X3)*Y1;
Yhat = X3*beta3;
Hrf_time_derv2 = Yhat;
subplot(3,3,4); imagesc([zscore(X3(:,1:2)) X3(:,3)]) ;colormap('gray');
subplot(3,3,[5 6]); plot(Y1,'r','LineWidth',3); grid on; axis tight
hold on; plot(Yhat,'--','LineWidth',2); grid on; axis tight

% stats 1
SSeffect = norm(Yhat-mean(Yhat)).^2;
Residuals = Y1 - Yhat;
SSerror = norm(Residuals-mean(Residuals)).^2;
R2 = SSeffect / SStotal;
df = rank(X3)-1;
dfe = length(Y1)-rank(X3);
```

```
F = (SSeffect/df) / (SSerror/dfe);
p_val = 1-spm_Fcdf(F,df,dfe);
mytitle = sprintf('condition1=%g and constant=%g \n R^2=%g F(%g,%g)=%g p=%g', beta3(1),
beta3(3), R2, df, dfe, F, p_val);
title(mytitle,'FontSize',14)

% stats 2
P = X3*pinv(X3); % H matrix
R = eye(size(Y1,1)) - P;
variance = ((R*Y1)'*(R*Y1)) / (size(Y1,1)-rank(X3));
C = [1 0 0];
T_con = (C*beta3) ./ sqrt(variance.*(C*pinv(X3'*X3)*C'));
p_con = 2*(1-spm_Tcdf(T_con, (size(Y1,1)-rank(X3))));
mytitle = sprintf('C = [1 0 0] T=%g p=%g', T_con, p_con);
subplot(3,3,4);title(mytitle,'FontSize',14)
```

Derivative orthogonal to [hrf regressor constant]

```
x = spm_orth2(SS);
X4 = [x(1:400,:) ones(400,1)];
beta4 = pinv(X4)*Y1;
Hrf_time_derv3 = Yhat;
subplot(3,3,7); imagesc([zscore(X4(:,1:2)) X4(:,3)]) ;colormap('gray');
subplot(3,3,[8 9]); plot(Y1,'r','LineWidth',3); grid on; axis tight
hold on; plot(Yhat,'--','LineWidth',2); grid on; axis tight

% stats 1
SSeffect = norm(Yhat-mean(Yhat)).^2;
Residuals = Y1 - Yhat;
SSerror = norm(Residuals-mean(Residuals)).^2;
R2 = SSeffect / SStotal;
df = rank(X4)-1;
dfe = length(Y1)-rank(X4);
F = (SSeffect/df) / (SSerror/dfe);
p_val = 1-spm_Fcdf(F,df,dfe);
mytitle = sprintf('condition1=%g constant=%g \n R^2=%g F(%g,%g)=%g p=%g', beta4(1), beta4(3),
R2, df, dfe, F, p_val);
title(mytitle,'FontSize',14)

% stats 2
P = X4*pinv(X4); % H matrix
R = eye(size(Y1,1)) - P;
variance = ((R*Y1)'*(R*Y1)) / (size(Y1,1)-rank(X4));
C = [1 0 0];
T_con = (C*beta4) ./ sqrt(variance.*(C*pinv(X4'*X4)*C'));
p_con = 2*(1-spm_Tcdf(T_con, (size(Y1,1)-rank(X4))));
mytitle = sprintf('C = [1 0 0] T=%g p=%g', T_con, p_con);
subplot(3,3,7); title(mytitle,'FontSize',14)
```

```
% additional figures to look at the hrf fit
figure('Name','hrf+derivative fit')
plot(Y1,'r','LineWidth',3); grid on; axis tight; hold on;
plot(X1*beta1,'k--','LineWidth',2); grid on; axis tight
plot(X2(:,[1 3])*beta2([1 3]),'--','LineWidth',2); grid on; axis tight
plot(X3(:,[1 3])*beta3([1 3]),'--g','LineWidth',2); grid on; axis tight
plot(X4(:,[1 3])*beta4([1 3]),'--m','LineWidth',2); grid on; axis tight
legend('Data','Hrf only','Model 1','Model 2','Model 3','Location','NorthEastOutside')
title('Plot of all models','FontSize',14)
```

See figure 5 in manuscript

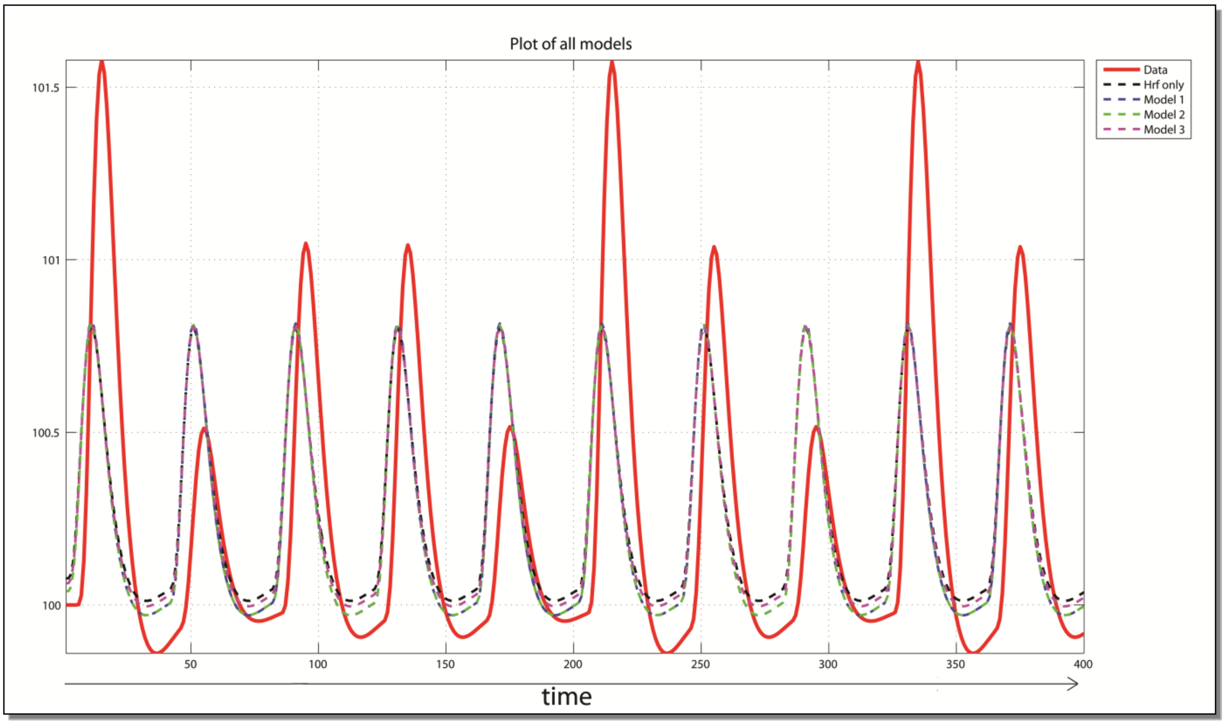

Supplementary Figure 2. Simulated data (red) and modelled hrf regressors. In black is showed the modelled response using the hrf only. In blue, green and purple is showed the part of the modelled response attributed to the hrf regressor when the model also include the 1st derivative (non orthogonalized = model 1 in blue, orthogonalized against the hrf regressor = model 2 in green and orthogonalized against the hrf regressor and the constant = model 3 in purple).

Redo the above analyses to look at the effect on parameter estimates

Shift the signal in time in a continuous fashion

```
index = 1;
for shift = -4:4

    Y1 = zeros(500,1); % 250 sec sample at 2Hz
    X1 = zeros(500,1);
    for i=1:10
        try
            Y1(onsets(i)+shift*2) = scale(i); % shift
        catch
            Y1(onsets(i)) = scale(i); % no shift - occurs for neg value at Y(1)
        end
        X1(onsets(i)) = 1;
    end

    SS(:,1) = conv(X1,xBF.bf(:,1));
    SS(:,2) = conv(X1,xBF.bf(:,2));
    X1 = [SS(1:400,1) ones(400,1)];
    Y1 = conv(Y1,spm_hrf(0.5));
    Y1 = Y1(1:400)+100;
    beta1 = pinv(X1)*Y1;
    hrf(1,index) = beta1(1);
    % No orthogonalization
    X2 = [SS(1:400,:) ones(400,1)];
    beta2 = pinv(X2)*Y1;
    hrf(2,index) = beta2(1);
    % Derivative orthogonal to hrf regressor
    x = spm_orth(SS);
    X3 = [x(1:400,:) ones(400,1)];
    beta3 = pinv(X3)*Y1;
    hrf(3,index) = beta3(1);
    % Derivative orthogonal to [hrf regressor constant]
    x = spm_orth2(SS);
    X4 = [x(1:400,:) ones(400,1)];
    beta4 = pinv(X4)*Y1;
    hrf(4,index) = beta4(1);

    % update index
    index = index+1;
end

figure('Name','Hrf estimates per orthogonalization procedure')
subplot(1,2,1); plot([-4:4],hrf(1,:),'c--','LineWidth',3); hold on
plot([-4:4],hrf([2 3 4],:),'LineWidth',3); axis square; grid on
legend('Hrf only','Model 1','Model 2','Model 3','Location','South')
title('Parameter estimates','FontSize',14)
subplot(1,2,2); D = [[hrf(2,:)-hrf(1,:)];[hrf(3,:)-hrf(1,:)];[hrf(4,:)-hrf(1,:)]];
plot([-4:4],D,'LineWidth',3); axis square; grid on
legend('Model 1','Model 2','Model 3','Location','NorthWest')
title('Distance to hrf only','FontSize',14)
```

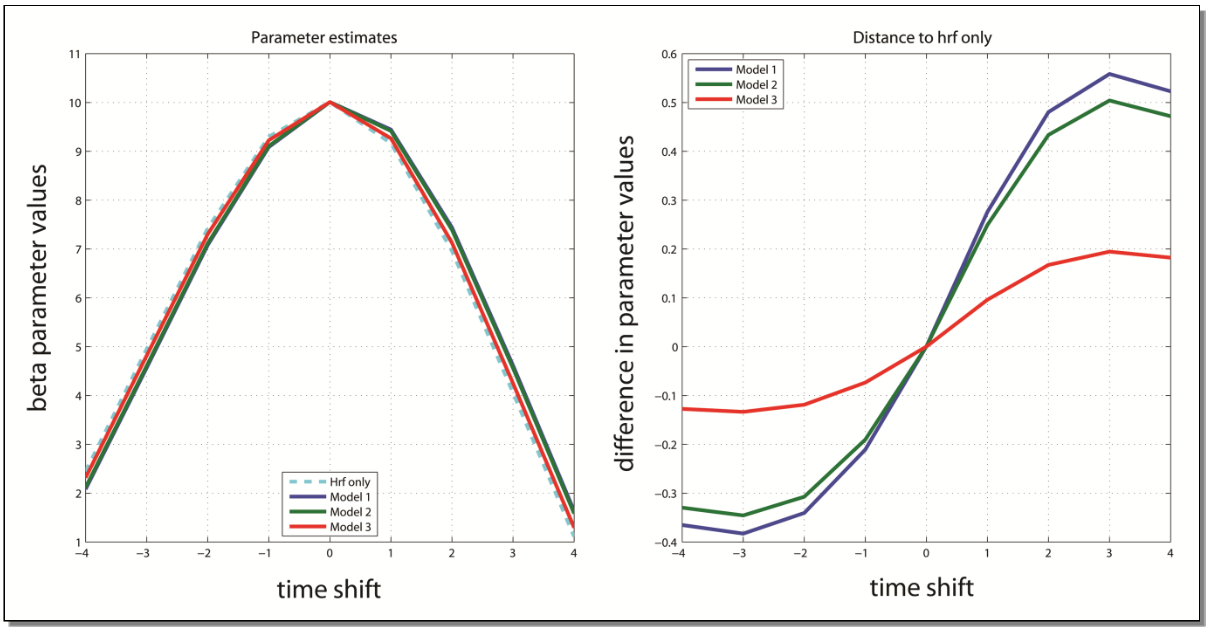

Supplementary Figure 3. Illustration of the difference in parameter values of the hrf regressor between a model including the hrf only and models including the 1st derivative (non orthogonalized = model 1 in blue, orthogonalized against the hrf regressor = model 2 in green and orthogonalized against the hrf regressor and the constant = model 3 in red).
