# Supplementary material for: Misconceptions in the use of the General Linear Model applied to functional MRI: a tutorial for junior neuro-imagers
Source: Front Neurosci. 2014 Jan 21;8:1. doi: 10.3389/fnins.2014.00001 (PMC3896880; doi:10.3389/fnins.2014.00001)
Supplement: Supplementary file 1 [file DataSheet1.ZIP › Annex 4.pdf]

Annex 4

To provide further insight into orthogonalization and how it impacts on parameter estimates and statistics, additional simulations were run. Parameter estimates and t values were computed on data simulating the same single condition periodic design as before but testing for a temporal shift of 0, +1 and +2 sec while varying systematically the inter-stimulus interval (isi) from 5 to 40 sec. This allowed testing, as a function of the correlation between the hrf and the constant term (since the longer the isi, the smaller the correlation), how much the parameter estimates for the different orthogonalization procedure differ from the hrf only model.

As illustrated supplementary figure 4, orthogonalization of the derivative against the hrf regressor and constant together, gives estimates identical to the hrf alone model. This is explained by the fact that the derivative is orthogonal to the whole design matrix (i.e. the subspace on which data are projected is simply 'augmented') and thus does not influence estimates. Other models gave closer estimates when the ISI was large, i.e. when the correlation between the hrf regressor and the constant term was low. Interestingly, while no orthogonalization or orthogonalization against the hrf regressor gave different parameter estimates, their corresponding T values were more similar to orthogonalization of the derivative against the hrf regressor and constant together. The reason for this discrepancy between relatively large parameter estimate differences vs. smaller T value differences is that T values are adjusted for the covariance between regressors (see equation 4 in the manuscript). All models, no matter the orthogonalization technique, give the same data fit (see figure 5), which means they have the same variance. The covariance however varies with the correlation between regressors. Orthogonalization procedures thus lead to different parameter estimates (which will impact on 2nd level analysis) but at the subject level, it impacts minimally on the statistics.

Orthogonalization effect

Simulation of event related designs analyzed using hrf+derivatives to show the effect of orthogonalization on parameter estimates

```
clear

% get the hrf and derivative
xBF.dt = 0.5;
xBF.name = 'hrf (with time derivative)';
xBF.length = 32;
xBF.order = 1;
xBF = spm_get_bf(xBF);
```

Simple periodic design

\-

```
index1 = 1;
scale = [15 5 10 10 5 15 10 5 15 10];

for shift = 0:2:4 % ie 0 +1 +2sec
```

```
    index2 = 1;

    for isi=1:8 % fron 5sec isi to 40sec
        onsets = [1:10*isi:100*isi];
        Y1 = zeros(50*isi,1); % sampling at 2Hz
        X1 = zeros(50*isi,1);
        for i=1:10
            Y1(onsets(i)+shift) = scale(i);
            X1(onsets(i)) = 1;
        end
        Y1 = conv(Y1,spm_hrf(0.5));

        clear SS
        SS(:,1) = conv(X1,xBF.bf(:,1));
        SS(:,2) = conv(X1,xBF.bf(:,2));
        L=min(length(SS),length(Y1));
        Y1 = Y1(1:L)+100;

        % --> design matrix with hrf only
        % -------------------------------
        X1 = [SS(1:L,1) ones(L,1)];
        beta1 = pinv(X1)*Y1;
        R = eye(size(Y1,1)) - (X1*pinv(X1));
        variance = ((R*Y1)'*(R*Y1)) / (size(Y1,1)-rank(X1));
        C = [1 0]; T(index1,index2,1) = (C*beta1) ./ sqrt(variance.*(C*pinv(X1'*X1)*C'));
        T_var(index1,index2,1) = variance; T_cov(index1,index2,1) = (C*pinv(X1'*X1)*C');

        % --> design matrix with derivatives
        % ----------------------------------
        X2 = [SS(1:L,:) ones(L,1)];
        beta2 = pinv(X2)*Y1;
        R = eye(size(Y1,1)) - (X2*pinv(X2));
        variance = ((R*Y1)'*(R*Y1)) / (size(Y1,1)-rank(X2));
        C = [1 0 0]; T(index1,index2,2) = (C*beta2) ./ sqrt(variance.*(C*pinv(X2'*X2)*C'));
        T_var(index1,index2,2) = variance; T_cov(index1,index2,2) = (C*pinv(X2'*X2)*C');

        % --> derivatives forced to be orthogonal to hrf
        % ----------------------------------------------------------
        x = spm_orth(SS);
        X3 = [x(1:L,:) ones(L,1)];
        beta3 = pinv(X3)*Y1;
        R = eye(size(Y1,1)) - (X3*pinv(X3));
        variance = ((R*Y1)'*(R*Y1)) / (size(Y1,1)-rank(X3));
        C = [1 0 0]; T(index1,index2,3) = (C*beta3) ./ sqrt(variance.*(C*pinv(X3'*X3)*C'));
        T_var(index1,index2,3) = variance; T_cov(index1,index2,3) = (C*pinv(X3'*X3)*C');

        % --> derivatives forced to be orthogonal to [hrf constant]
        % ----------------------------------------------------------
        x = spm_orth2(SS);
        X4 = [x(1:L,:) ones(L,1)];
        beta4 = pinv(X4)*Y1;
        R = eye(size(Y1,1)) - (X4*pinv(X4));
        variance = ((R*Y1)'*(R*Y1)) / (size(Y1,1)-rank(X4));
        C = [1 0 0]; T(index1,index2,4) = (C*beta4) ./ sqrt(variance.*(C*pinv(X4'*X4)*C'));
        T_var(index1,index2,4) = variance; T_cov(index1,index2,4) = (C*pinv(X4'*X4)*C');

        % record beta(1) parameters + correlations between regressors
```

```
        % -----------------------------------------------------------
        data(index1,index2,:) = [beta1(1) beta2(1) beta3(1) beta4(1)];
        % correlation hrf / ones
        cos_theta1(index1,index2) =  (dot(X1(:,1),X1(:,2))) / (norm(X1(:,1))*norm(X1(:,2)));
        % correlation derivative / ones
        cos_theta2(index1,index2) =  (dot(SS(1:L,2),X1(:,2))) /
(norm(SS(1:L,2))*norm(X1(:,2)));
        index2 = index2+1;
    end
    index1 = index1+1;
end

% check the behaviour of T values
% all model provide the same data fit so the variance should be the same
% only covariance increases
% 1 - check there is a unique variance value for all models
% 2 - check that on average the covariance is higher in models 2 and 3
for shift = 1:3
    test(shift) = mean(mean(single(squeeze(T_var(shift,:,2:4))) ==
single(repmat(mean(unique(squeeze(T_var(shift,:,2:4))','rows'))',1,3))));
    test2(shift,:) = mean(squeeze(T_cov(shift,:,:)));
end

if sum(test) == 3
    fprintf('all model have the same variance \n');
end

test2 = unique(repmat(test2(:,1),1,3) - test2(:,2:4));
for model = 1:3
    if test2(model) < 0
        fprintf('but covariance of model %g is larger of %g \n',model,abs(test2(model)))
    elseif test2(model) == 0
        fprintf('and covariance of model %g is identical as hrf only \n',model)
    end
end
```

```
all model have the same variance
but covariance of model 1 is larger of 0.0179129
but covariance of model 2 is larger of 0.0147553
and covariance of model 3 is identical as hrf only
```

```
% figure
s = [0 1 2]; % shift in sec
isi= [5:5:40]; % isi in sec
figure; index = 1;
for shift = 1:3
    subplot(3,4,index);
    plot(isi,squeeze(data(shift,:,1)),'LineWidth',3);
    grid on; hold on;
    plot(isi,squeeze(data(shift,:,2)),'k','LineWidth',3);
    plot(isi,squeeze(data(shift,:,3)),'--g','LineWidth',3);
    plot(isi,squeeze(data(shift,:,4)),'--r','LineWidth',3);
    ylabel('beta parameters','Fontsize',14);
    xlabel('ISI','Fontsize',14); axis tight
    title(['shift of ' num2str(s(shift)) ' sec'],'Fontsize',14);
```

```
    subplot(3,4,index+1);
    plot(fliplr(cos_theta1(1,:)),squeeze(data(shift,:,1)),'LineWidth',3);
    grid on; hold on;
    plot(fliplr(cos_theta1(1,:)),squeeze(data(shift,:,2)),'k','LineWidth',3);
    plot(fliplr(cos_theta1(1,:)),squeeze(data(shift,:,3)),'--g','LineWidth',3);
    plot(fliplr(cos_theta1(1,:)),squeeze(data(shift,:,4)),'--r','LineWidth',3);
    ylabel('beta parameters','Fontsize',14);
    xlabel('correlation hrf / constant','Fontsize',14); axis tight
    title(['shift of ' num2str(s(shift)) ' sec'],'Fontsize',14);
    legend('hrf only','no orth','spm orth','spm orth2')

    subplot(3,4,index+2);
    plot(fliplr(cos_theta1(1,:)),squeeze(T(shift,:,1)),'LineWidth',3);
    grid on; hold on;
    plot(fliplr(cos_theta1(1,:)),squeeze(T(shift,:,2)),'k','LineWidth',3);
    plot(fliplr(cos_theta1(1,:)),squeeze(T(shift,:,3)),'--g','LineWidth',3);
    plot(fliplr(cos_theta1(1,:)),squeeze(T(shift,:,4)),'--r','LineWidth',3);
    ylabel('T values','Fontsize',14);
    xlabel('correlation hrf / constant','Fontsize',14); axis tight
    title(['shift of ' num2str(s(shift)) ' sec'],'Fontsize',14);

    subplot(3,4,index+3);
    tmp = sortrows([squeeze(data(shift,:,1))' squeeze(T(shift,:,1))']);
    plot(tmp(:,1),tmp(:,2),'LineWidth',3);
    grid on; hold on;
    tmp = sortrows([squeeze(data(shift,:,2))' squeeze(T(shift,:,2))']);
    plot(tmp(:,1),tmp(:,2),'k','LineWidth',3);
    tmp = sortrows([squeeze(data(shift,:,3))' squeeze(T(shift,:,3))']);
    plot(tmp(:,1),tmp(:,2),'--g','LineWidth',3);
    tmp = sortrows([squeeze(data(shift,:,4))' squeeze(T(shift,:,4))']);
    plot(tmp(:,1),tmp(:,2),'--r','LineWidth',3);
    ylabel('T values','Fontsize',14);
    xlabel('parameter estimate','Fontsize',14);
    axis([min(min(data(shift,:,:))) max(max(data(shift,:,:))) 7.8 66.8])
    title(['shift of ' num2str(s(shift)) ' sec'],'Fontsize',14);
    index= index+4;
end
```

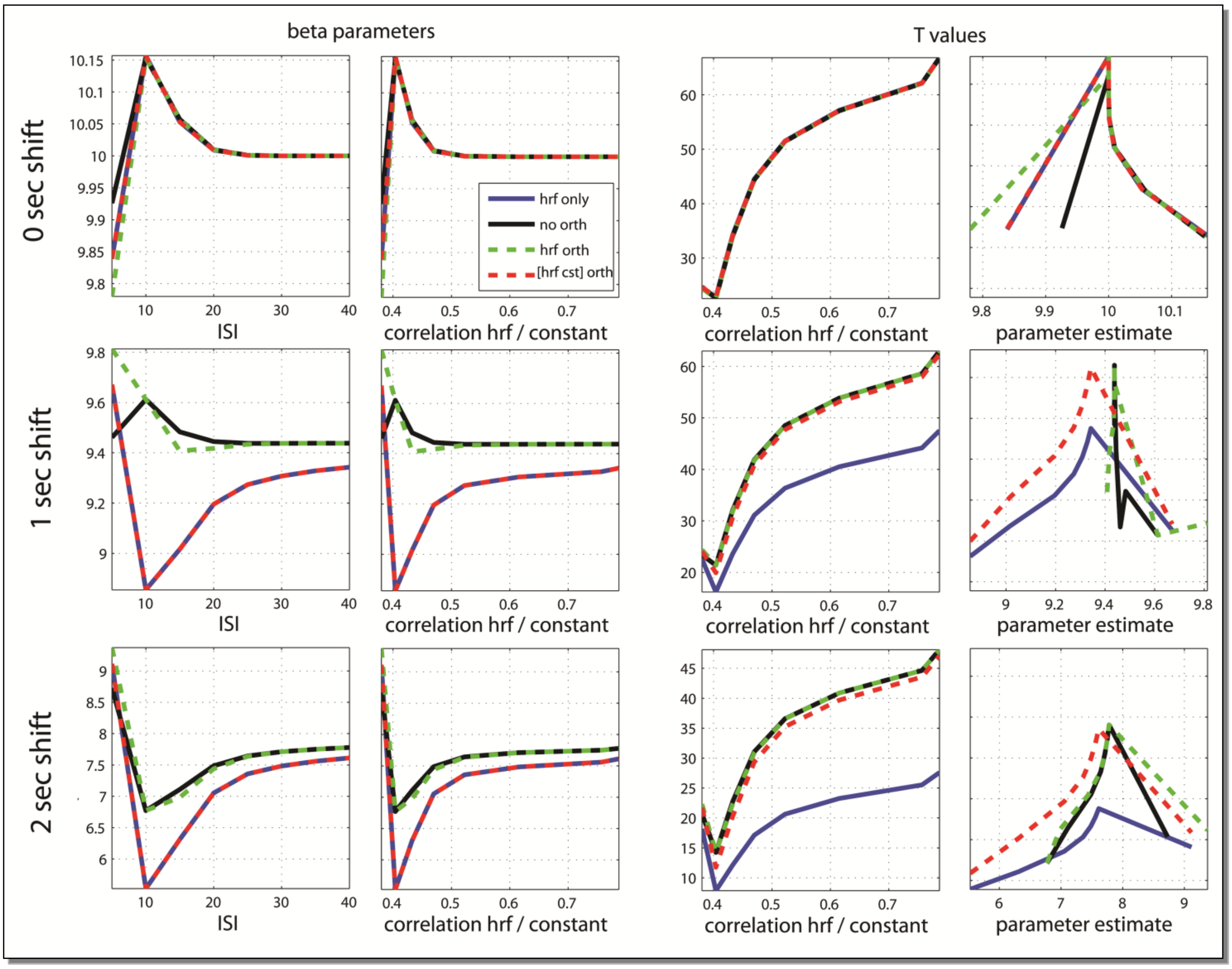

Supplementary Figure 1. Results of the parameter estimates and T values are presented for 0 sec shift (top), 1 sec shift (middle) and 2sec shift (bottom). On the left parameter estimate values are plotted both as a function of the ISI and as a function of the correlation between the hrf and constant. As illustrated, the relationship is almost one to one between ISI and correlation such as the plots are very similar. On the right T values are plotted as a function of the correlation between the hrf and constant and as a function of the parameter estimate values. This shows that while T values vary as a monotonic function from r=0.1 to 1, T values changes do not follow the strength of the parameter estimates.
