# Supplementary material for: Misconceptions in the use of the General Linear Model applied to functional MRI: a tutorial for junior neuro-imagers
Source: Front Neurosci. 2014 Jan 21;8:1. doi: 10.3389/fnins.2014.00001 (PMC3896880; doi:10.3389/fnins.2014.00001)
Supplement: Supplementary file 1 [file DataSheet1.ZIP › Annex 5.pdf]

Annex 5

PSC_simulations

This Matlab code, is a simple demonstration of how to compute percentage signal change using the GLM parameter estimates. It also compares this approach to computing the PSC from the modelled data.

```
clear all

% hrf model using SPM function
% ---------------------------
xBF.dt = 0.5;
xBF.name = 'hrf (with time derivative)';
xBF.length = 32;
xBF.order = 1;
xBF = spm_get_bf(xBF);
```

Simulate a Periodic design

____

```
% create a hrf = brain response and scale
scale = [15 5 10 10 5 15 10 5 15 10];

% create a periodic design
% ------------------------
onsets = 1:40:400;
Y1 = zeros(500,1); % 250 sec sample at 2Hz
X1 = zeros(500,1);
for i=1:10
    Y1(onsets(i)) = scale(i);
    X1(onsets(i)) = 1;
end
```

PSC from GLM parameters for the original data

____

```
SS = conv(X1,spm_hrf(0.5));
X1 = [SS(1:400) ones(400,1)];
Y1 = conv(Y1,spm_hrf(0.5));
Y1 = Y1(1:400)+100;
Average_signal(1) = mean(Y1); % average of the time series
SStotal = norm(Y1-mean(Y1)).^2;
beta1 = pinv(X1)*Y1;
Yhat = X1*beta1;

% start the figure
figure('Name','Fig. 6. Super sampled vs downsampled designs');
subplot(3,4,1); imagesc([zscore(X1(:,1)) X1(:,2)]);colormap('gray');
subplot(3,4,2); plot(Y1,'r','LineWidth',3); grid on; axis tight
hold on; plot(Yhat,'--','LineWidth',2); grid on; axis tight
```

```
% stats 1
SSeffect = norm(Yhat-mean(Yhat)).^2;
Residuals = Y1 - Yhat;
SSerror = norm(Residuals-mean(Residuals)).^2;
R2 = SSeffect / SStotal;
df = rank(X1)-1;
dfe = length(Y1)-rank(X1);
F = (SSeffect/df) / (SSerror/dfe);
p_val = 1-spm_Fcdf(F,df,dfe);
mytitle = sprintf('R^2=%g \n F(%g,%g)=%g p=%g', R2, df, dfe, F, p_val);
title(mytitle,'FontSize',14)

% stats 2
P = X1*pinv(X1);
R = eye(size(Y1,1)) - P;
variance = ((R*Y1)'*(R*Y1)) / (size(Y1,1)-rank(X1));
C = [1 0];
T_con = (C*beta1) ./ sqrt(variance.*(C*pinv(X1'*X1)*C'));
p_con = 2*(1-spm_Tcdf(T_con, (size(Y1,1)-rank(X1))));

% -------------------------
% percentage signal change
% -------------------------
% since all events have the same size, we can take the max as SF
myrange(1) = max(X1(:,1));
PSC1 = beta1(1)*max(X1(:,1))./beta1(2).*100;
mytitle = sprintf('T=%g p=%g PSC=%g%%  \n', T_con, p_con, PSC1(1));
subplot(3,4,1); title(mytitle,'FontSize',14)
subplot(3,4,[9 10]); plot(X1(:,1),'LineWidth',3); grid on;
title(['periodic regressor max' num2str(max(X1(:,1)))],'FontSize',14)

% lets' compare with the SF being the 'standard' trial
% either use the height as SF
event = xBF.bf(:,1);
PSC1b = beta1(1)*max(event)./beta1(2).*100;

% or use a model of the response - MarsBar style
scaled_event = event * beta1(1);
PSC_Yhat = max(scaled_event) / beta1(2) * 100;

% for comparison, compute PSC from for the original data
MY = reshape(Y1,40,10); % bin data per event
PSC_Y = mean(max(MY)) - 100; % 100 is the real baseline

% print output
% the small difference between real and modelled PSC comes from the
% constant beta1(2) which is estimated a little below 100 - note that for
% this simple periodic design, all estimates are the same
fprintf('PSC relative to baseline in the data = %g \n',PSC_Y)
fprintf('PSC using GLM parameters and SF from X = %g \n',PSC1)
fprintf('PSC using GLM parameters and SF from standard trial = %g \n',PSC1)
fprintf('PSC using modelled trial (MarsBaR) = %g \n',PSC_Yhat)
```

```
PSC relative to baseline in the data = 1.04363
      PSC using GLM parameters and SF from X = 1.05349
      PSC using GLM parameters and SF from standard trial = 1.05349
      PSC using modelled trial (MarsBaR) = 1.05349
```

PSC from GLM parameters for downsampled data - as for real fMRI

```
X1 = X1(1:4:400,:); % downsample
Y1 = Y1(1:4:400);   % downsample
Average_signal(2) = mean(Y1);
SStotal = norm(Y1-mean(Y1)).^2;

beta1 = pinv(X1)*Y1;
Yhat = X1*beta1;
subplot(3,4,3); imagesc([zscore(X1(:,1)) X1(:,2)]);colormap('gray');
subplot(3,4,4); plot(Y1,'r','LineWidth',3); grid on; axis tight
hold on; plot(Yhat,'--','LineWidth',2); grid on; axis tight

% stats 1
SSeffect = norm(Yhat-mean(Yhat)).^2;
Residuals = Y1 - Yhat;
SSerror = norm(Residuals-mean(Residuals)).^2;
R2 = SSeffect / SStotal;
df = rank(X1)-1;
dfe = length(Y1)-rank(X1);
F = (SSeffect/df) / (SSerror/dfe);
p_val = 1-spm_Fcdf(F,df,dfe);
mytitle = sprintf('R^2=%g \n F(%g,%g)=%g p=%g', R2, df, dfe, F, p_val);
title(mytitle,'FontSize',14)

% stats 2
P = X1*pinv(X1); % H matrix
R = eye(size(Y1,1)) - P;
variance = ((R*Y1)'*(R*Y1)) / (size(Y1,1)-rank(X1));
C = [1 0]; % contrast for condition 2 > condition 1
T_con = (C*beta1) ./ sqrt(variance.*(C*pinv(X1'*X1)*C')); % T value
p_con = 2*(1-spm_Tcdf(T_con, (size(Y1,1)-rank(X1))));

% -------------------------
% percentage signal change
% -------------------------
% for direct comparison of the effect of sampling, and all events still have
% the same height, let's take the max
myrange(2) = max(X1(:,1));
PSC(1) = beta1(1)*max(X1(:,1))./beta1(2).*100 ; % scaling using downsampled design

% as before, compare with SF taken from the super sampled standard trial
myrange(3) = max(event);
PSC(2) = beta1(1)*max(event)./beta1(2).*100;
mytitle = sprintf('T=%g p=%g PSC=%g%% \n PSC using original X=%g%%', T_con, p_con,
PSC(1),PSC(2));
subplot(3,4,3); title(mytitle,'FontSize',14)

% or even compare with the modelled version (MarsBar style)
scaled_event = event * beta1(1);
```

```
PSC_Yhat = max(scaled_event) / beta1(2) * 100;

% print out the results
fprintf('PSC using GLM parameters from downsampled design = %g \n',PSC(1))
fprintf('PSC using GLM parameters from downsampled deisgn but with the SF from the standard
trial= %g \n',PSC(2))
fprintf('PSC using modelled trial (MarsBaR) = %g \n',PSC_Yhat)
```

```
PSC using GLM parameters from downsampled design = 0.963621
PSC using GLM parameters from downsampled deisgn but with the SF from the standard trial=
1.05349
PSC using modelled trial (MarsBaR) = 1.05349
```

Fast event related design modelling the same 'brain responses' as before

```
onsets = [1 31 81 93 161 201 218 291 321 361];
Y2 = zeros(500,1); % 250 sec sample at 2Hz
X2 = zeros(500,1);
for i=1:10
    Y2(onsets(i)) = scale(i);
    X2(onsets(i)) = 1;
end
```

PSC from GLM parameters for the original data

```
SS = conv(X2,spm_hrf(0.5));
X2 = [SS(1:400) ones(400,1)];
Y2 = conv(Y2,spm_hrf(0.5));
Y2 = Y2(1:400)+100;
Average_signal(3) = mean(Y2);
SStotal = norm(Y2-mean(Y2)).^2;

beta2 = pinv(X2)*Y2;
Yhat = X2*beta2;
subplot(3,4,5); imagesc([zscore(X2(:,1)) X2(:,2)]);colormap('gray');
subplot(3,4,6); plot(Y2,'r','LineWidth',3); grid on; axis tight
hold on; plot(Yhat,'--','LineWidth',2); grid on; axis tight

% stats 1
SSeffect = norm(Yhat-mean(Yhat)).^2;
Residuals = Y2 - Yhat;
SSerror = norm(Residuals-mean(Residuals)).^2;
R2 = SSeffect / SStotal;
df = rank(X2)-1;
dfe = length(Y2)-rank(X2);
F = (SSeffect/df) / (SSerror/dfe);
p_val = 1-spm_Fcdf(F,df,dfe);
mytitle = sprintf('R^2=%g \n F(%g,%g)=%g p=%g \n', R2, df, dfe, F, p_val);
title(mytitle,'FontSize',14)

% stats 2
```

```
P = X2*pinv(X2); % H matrix
R = eye(size(Y2,1)) - P;
variance = ((R*Y2)'*(R*Y2)) / (size(Y2,1)-rank(X2));
C = [1 0]; % contrast for condition 2 > condition 1
T_con = (C*beta2) ./ sqrt(variance.*(C*pinv(X2'*X2)*C')); % T value
p_con = 2*(1-spm_Tcdf(T_con, (size(Y2,1)-rank(X2))));

% -------------------------
% percentage signal change
% -------------------------
% what is happening if we use the SF from the current design
myrange(4) = max(SS);
PSC2     = beta2(1).* max(SS)./beta2(2).*100 ;

% consider instred using the same SF across design, by taking the standard
% event height as SF
PSC2cor = beta2(1).* max(event) ./ beta2(2).*100 ; % ie scale by max of a single event
mytitle = sprintf('T=%g p=%g PSC=%g%% \n PSC corrected=%g%% \n', T_con, p_con,
PSC2(1),PSC2cor);
subplot(3,4,5); title(mytitle,'FontSize',14)
subplot(3,4,[11 12]); plot(X2(:,1),'LineWidth',3); grid on;
title('periodic regressor max 0.115','FontSize',14)

% do the same MarsBaR style - gives the same result
scaled_event = event * beta2(1);
PSC_Yhat = max(scaled_event) / beta2(2) * 100;

% finally, for compaison, get the PSC from for data
% get the max of event event
for e=1:10
    index = onsets(e);
    if e < 10
        index2 = onsets(e+1);
        max_Y2(e) = max(Y2(index:index2));
    else
        max_Y2(e) = max(Y2(index:end));
    end
end
PSC_Y = mean(max_Y2) - 100;

% print output
fprintf('PSC relative to baseline in the data = %g \n',PSC_Y)
fprintf('PSC using GLM parameters and SF from X = %g \n',PSC2)
fprintf('PSC using GLM parameters and SF from standard trial= %g \n',PSC2cor)
fprintf('PSC using modelled event (MarsBaR) = %g \n',PSC_Yhat)
```

```
PSC relative to baseline in the data = 1.04109
      PSC using GLM parameters and SF from X = 1.1506
      PSC using GLM parameters and SF from standard trial= 1.04696
      PSC using modelled event (MarsBaR) = 1.04696
```

PSC from GLM parameters for downsampled data - as for real fMRI

```
X2 = X2(1:4:400,:);
Y2 = Y2(1:4:400);
Average_signal(4) = mean(Y2);
SStotal = norm(Y2-mean(Y2)).^2;

beta2 = pinv(X2)*Y2;
Yhat = X2*beta2;
subplot(3,4,7); imagesc([zscore(X2(:,1)) X2(:,2)]);colormap('gray');
subplot(3,4,8); plot(Y2,'r','LineWidth',3); grid on; axis tight
hold on; plot(Yhat,'--','LineWidth',2); grid on; axis tight

% stats 1
SSeffect = norm(Yhat-mean(Yhat)).^2;
Residuals = Y2 - Yhat;
SSerror = norm(Residuals-mean(Residuals)).^2;
R2 = SSeffect / SStotal;
df = rank(X2)-1;
dfe = length(Y2)-rank(X2);
F = (SSeffect/df) / (SSerror/dfe);
p_val = 1-spm_Fcdf(F,df,dfe);
mytitle = sprintf('R^2=%g \n F(%g,%g)=%g p=%g \n', R2, df, dfe, F, p_val);
title(mytitle,'FontSize',14)

% stats 2
P = X2*pinv(X2); % H matrix
R = eye(size(Y2,1)) - P;
variance = ((R*Y2)'*(R*Y2)) / (size(Y2,1)-rank(X2));
C = [1 0]; % contrast for condition 2 > condition 1
T_con = (C*beta2) ./ sqrt(variance.*(C*pinv(X2'*X2)*C'));
p_con = 2*(1-spm_Tcdf(T_con, (size(Y2,1)-rank(X2))));

% -------------------------
% percentage signal change
% -------------------------
% as before, what if we take SF from the current design
myrange(5) = max(X2(:,1));
PSC2(1)    = beta2(1).* max(X2(:,1))./beta2(2).*100 ;

% by comparison, use the SF from the Super Sampled design
myrange(6) = max(SS);
PSC2(2)    = beta2(1)./beta2(2).*100 .* max(SS); % max of the super sampled design

% now use the SF from the standard trial
PSC2cor = beta2(1).*max(event)./beta2(2).*100;
mytitle = sprintf('T=%g p=%g PSC=%g%% \n PSC using original X=%g%% \n PSC corrected=%g%%',
T_con, p_con, PSC2(1),PSC2(2),PSC2cor);
subplot(3,4,7); title(mytitle,'FontSize',14)

% print output
fprintf('PSC using GLM parameters = %g \n',PSC2(1))
fprintf('PSC using GLM parameters but SF from the super sampled design = %g \n',PSC2(2))
fprintf('PSC using GLM parameters but SF from the standard trial = %g \n',PSC2cor)
```

```
% final check
fprintf('Average values in each simulation %g %g %G %g
\n',Average_signal(1),Average_signal(2),Average_signal(3),Average_signal(4))

% ----------------------------
% Treat for SPM users:
% It is easy to recreate the super-sampled design by 1 - loading a SPM.mat
% then 2. define the basis functions used bf = SPM.xBF.bf;
% VolteraValue = SPM.xBF.Volterra; and 3. read session-wise such as for a
% session s, we can retrieve onsets with U = spm_get_ons(SPM,s); and get
% the super sampled design with [X,Xn,Fc] = spm_Volterra(U,bf,VolteraValue);
%
% To get the SF from the standard trial simply get the basis function(s)
% xBF.dt = SPM.xBF.dt;
% xBF.name = SPM.xBF.name;
% xBF.length = SPM.xBF.length;
% xBF.order = SPM.xBF.order;
% xBF = spm_get_bf(xBF);
% SF = max(xBF.bf(:,1));
% ----------------------------
```

```
PSC using GLM parameters = 1.12388
PSC using GLM parameters but SF from the super sampled design = 1.15056
PSC using GLM parameters but SF from the standard trial = 1.04692
Average values in each simulation 100.251 100.251 100.251 100.251
```

See figure 6 in manuscript

Replicate the analyses adding a temporal shift in the data and the temporal derivative to the model

```
clear all

% hrf model using SPM function
% ---------------------------
xBF.dt = 0.5;
xBF.name = 'hrf (with time derivative)';
xBF.length = 32;
xBF.order = 1;
xBF = spm_get_bf(xBF);
```

Periodic design

```
scale = [15 5 10 10 5 15 10 5 15 10];

onsets = 1:40:400;
Y1 = zeros(500,1);
X1 = zeros(500,1);
for i=1:10
    Y1(onsets(i)+4) = scale(i);
    X1(onsets(i)) = 1;
end
Y1 = conv(Y1,spm_hrf(0.5));
```

```
Y1 = Y1(1:400)+100;
SS(:,1) = conv(X1,xBF.bf(:,1));
SS(:,2) = conv(X1,xBF.bf(:,2));
x = spm_orth2(SS);
X1 = [x(1:400,:) ones(400,1)];
Average_signal(1) = mean(Y1);
SStotal = norm(Y1-mean(Y1)).^2;

beta1 = pinv(X1)*Y1;
Yhat = X1*beta1;
figure('Name','Fig. 7. PSC using the 1st derivative');
subplot(2,3,1); imagesc([zscore(X1(:,1:2)) X1(:,2)]);colormap('gray');
subplot(2,3,2); plot(Y1,'r','LineWidth',3); grid on; axis tight
hold on; plot(X1(:,[1 3])*beta1([1 3]),'--g','LineWidth',2); grid on; axis tight
title('data and fit using the hrf only','Fontsize',14)
subplot(2,3,3); plot(Y1,'r','LineWidth',3); grid on; axis tight
hold on; plot(Yhat,'--','LineWidth',2); grid on; axis tight
title('data and fit using the hrf + derivative','Fontsize',14)

% -------------------------
% percentage signal change
% -------------------------
% if we do as before using hrf only
event = xBF.bf(:,1);
PSC1 = beta1(1)*max(event)./beta1(3).*100;

% but since we have a temporal derivative we can combine the bf
combined_event = xBF.bf(:,[1 2])*[1 1]';
H = (sqrt(((beta1(1)^2)*sum(X1(:,1).^2))+((beta1(2)^2)*sum(X1(:,2).^2))))*sign(beta1(1));
PSC1cor = H*max(combined_event)./beta1(3).*100 ;
mytitle = sprintf('PSC=%g%%  \n PSC adjusted=%g%%', PSC1, PSC1cor);
subplot(2,3,1); title(mytitle,'FontSize',14)

% do the same MarsBaR style - gives different
event = xBF.bf(:,1); scaled_event = event * beta1(1);
deriv = xBF.bf(:,2); scaled_deriv = deriv * beta1(2);
PSC_Yhat = max(scaled_event+scaled_deriv) / beta1(3) * 100;

% for comparison compute PSC in the data
% bin data per event
MY = reshape(Y1,40,10);
PSC_Y = mean(max(MY)) - 100; % 100 is the real baseline

% print output
% the MarsBaR approach assumes the hrf regressor is fine, but in fact it is
% biased (see Cahloun et al. 2004) hence the discrepency
fprintf('PSC in the data relative to baseline = %g \n',PSC_Y)
fprintf('PSC using GLM hrf information only = %g \n',PSC1)
fprintf('PSC using GLM parameters combined= %g \n',PSC1cor)
fprintf('PSC using modelled event (MarsBaR) = %g \n',PSC_Yhat)
```

```
PSC in the data relative to baseline = 1.04363
      PSC using GLM hrf information only = 0.749799
      PSC using GLM parameters combined= 1.04294
      PSC using modelled event (MarsBaR) = 0.979761
```

Fast event related with the same 'brain responses'

```
clear SS
onsets = [1 31 81 93  161 201 218 291 321 361];
Y2 = zeros(500,1); % 250 sec sample at 2Hz
X2 = zeros(500,1);
for i=1:10
    Y2(onsets(i)+4) = scale(i);
    X2(onsets(i)) = 1;
end
Y2 = conv(Y2,spm_hrf(0.5));
Y2 = Y2(1:400)+100;
SS(:,1) = conv(X2,xBF.bf(:,1));
SS(:,2) = conv(X2,xBF.bf(:,2));
x = spm_orth2(SS);
X2 = [x(1:400,:) ones(400,1)];

beta2 = pinv(X2)*Y2;
Yhat = X2*beta2;
subplot(2,3,4); imagesc([zscore(X2(:,1:2)) X2(:,2)]);colormap('gray');
subplot(2,3,5); plot(Y2,'r','LineWidth',3); grid on; axis tight
hold on; plot(X2(:,[1 3])*beta2([1 3]),'--g','LineWidth',2); grid on; axis tight
title('data and fit using the hrf only','Fontsize',14)
subplot(2,3,6); plot(Y2,'r','LineWidth',3); grid on; axis tight
hold on; plot(Yhat,'--','LineWidth',2); grid on; axis tight
title('data and fit using the hrf + derivative','Fontsize',14)

% -------------------------
% percentage signal change
% -------------------------
% use hrf only
PSC2 = beta2(1).* max(event)./beta2(3).*100;

% use hrf and derivative
H  = (sqrt(((beta2(1)^2)*sum(X2(:,1).^2))+((beta2(2)^2)*sum(X2(:,2).^2))))*sign(beta2(1));
PSC2cor = H*max(combined_event)./beta2(3).*100 ;
mytitle = sprintf('PSC=%g%%  \n PSC adjusted=%g%%', PSC2, PSC2cor);
subplot(2,3,4); title(mytitle,'FontSize',14)

% do the same MarsBaR style - gives the same result
scaled_event = event * beta2(1);
scaled_deriv = deriv * beta2(2);
PSC_Yhat = max(scaled_event+scaled_deriv) / beta2(3) * 100;

% compute PSC from the data
% get the max of event event
for e=1:10
    index = onsets(e);
    if e < 10
        index2 = onsets(e+1)+4;
```

```
        max_Y2(e) = max(Y2(index:index2));
    else
        max_Y2(e) = max(Y2(index:end));
    end
end
PSC_Y = mean(max_Y2) - 100;


% print output
fprintf('PSC in the data relative to baseline = %g \n',PSC_Y)
fprintf('PSC using GLM hrf information only = %g \n',PSC2)
fprintf('PSC using GLM parameters combined= %g \n',PSC2cor)
fprintf('PSC using modelled event (MarsBaR) = %g \n',PSC_Yhat)
```

```
PSC in the data relative to baseline = 1.07569
PSC using GLM hrf information only = 0.79311
PSC using GLM parameters combined= 1.04716
PSC using modelled event (MarsBaR) = 1.00529
```

test as well by down-sampling

```
onsets = 1:40:400;
Y1 = zeros(500,1); % 250 sec sample at 2Hz
X1 = zeros(500,1);
for i=1:10
    Y1(onsets(i)+4) = scale(i);
    X1(onsets(i)) = 1;
end
Y1 = conv(Y1,spm_hrf(0.5));
Y1 = Y1(1:400)+100;
SS(:,1) = conv(X1,xBF.bf(:,1));
SS(:,2) = conv(X1,xBF.bf(:,2));
x = spm_orth2(SS);
X1 = [x(1:400,:) ones(400,1)];
x1 = X1(1:4:400,:);
y1 = Y1(1:4:400);

beta1 = pinv(x1)*y1;
Yhat = x1*beta1;
figure; subplot(1,3,1); imagesc([zscore(x1(:,1:2)) x1(:,2)]);colormap('gray');
subplot(1,3,2); plot(y1,'r','LineWidth',3); grid on; axis tight
hold on; plot(x1(:,[1 3])*beta1([1 3]),'--g','LineWidth',2); grid on; axis tight
title('data and fit using the hrf only','Fontsize',14)
subplot(1,3,3); plot(y1,'r','LineWidth',3); grid on; axis tight
hold on; plot(Yhat,'--','LineWidth',2); grid on; axis tight
title('data and fit using the hrf + derivative','Fontsize',14)

% PSC - also need to work with the upsampled design
% use hrf information only
PSC1 = beta1(1)*max(event)./beta1(3).*100 ;

% use hrf and derivative
H  = (sqrt(((beta1(1)^2)*sum(X1(:,1).^2))+((beta1(2)^2)*sum(X1(:,2).^2))))*sign(beta1(1));
PSC1cor = H*max(combined_event)./beta1(3).*100 ;
mytitle = sprintf('PSC=%g%%  \n PSC adjusted=%g%%', PSC1, PSC1cor);
subplot(1,3,1); title(mytitle,'FontSize',14)
```

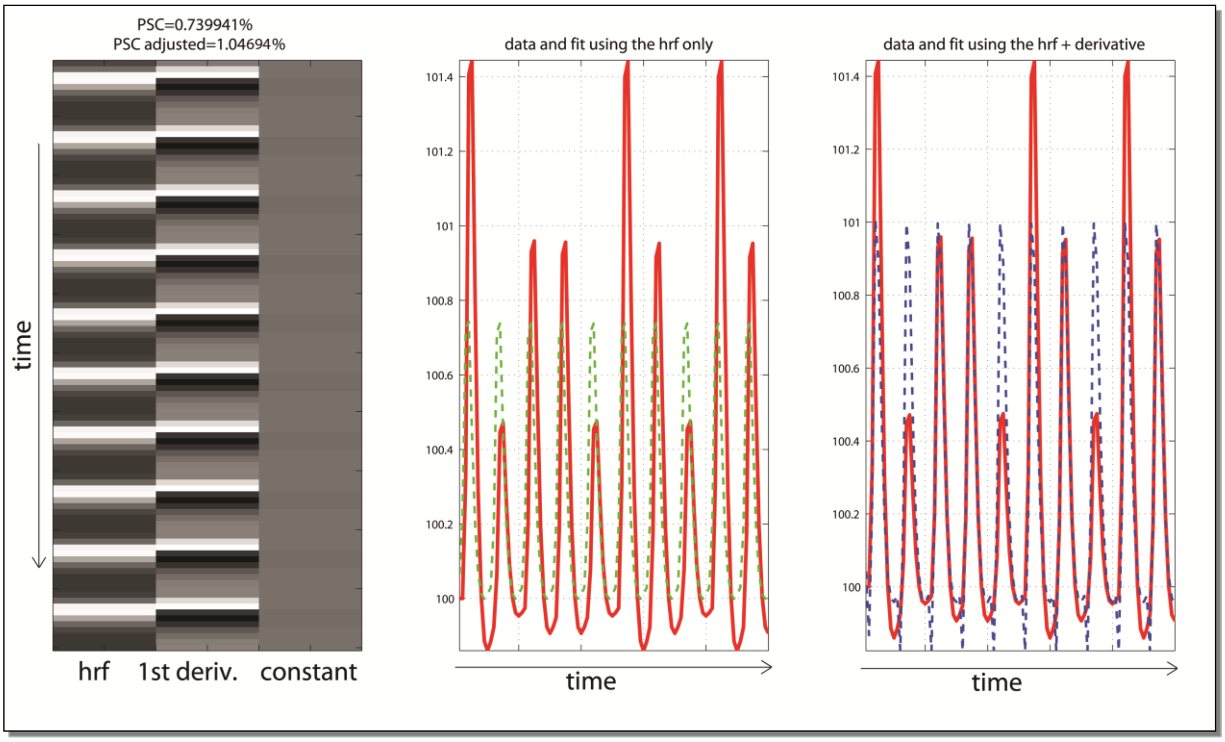

Supplementary Figure 5. Illustration of the PSC computed using the beta parameters for the hrf and derivative on down-sampled data.
